# Determining a musculoskeletal system’s pre-stretched state using continuum–mechanical forward modelling and joint range optimization

**DOI:** 10.1007/s10237-024-01821-x

**Published:** 2024-04-15

**Authors:** Okan Avci, Oliver Röhrle

**Affiliations:** 1 Fraunhofer Institute for Manufacturing Engineering and Automation IPA, Nobelstr. 12, 70569 Stuttgart, Germany; 2 Institute of Modelling and Simulation for Biomechanical Systems and Cluster of Excellence for Simulation Technology, University of Stuttgart, Pfaffenwaldring 5a, 70569 Stuttgart, Germany

**Keywords:** Pre-stretched joint system optimization, Elbow muscle–tendon-complex, Continuum–mechanical muscle model, Forward musculoskeletal system simulation, Computational finite element modelling, In silico orthopaedics

## Abstract

The subject-specific range of motion (RoM) of a musculoskeletal joint system is balanced by pre-tension levels of individual muscles, which affects their contraction capability. Such an inherent pre-tension or pre-stretch of muscles is not measureable with in vivo experiments. Using a 3D continuum mechanical forward simulation approach for motion analysis of the musculoskeletal system of the forearm with 3 flexor and 2 extensor muscles, we developed an optimization process to determine the muscle fibre pre-stretches for an initial arm position, which is given human dataset. We used RoM values of a healthy person to balance the motion in extension and flexion. The performed sensitivity study shows that the fibre pre-stretches of the *m. brachialis*, *m. biceps brachii* and *m. triceps brachii* with $91\%$ dominate the objective flexion ratio, while *m. brachiradialis* and *m. anconeus* amount $7.8\%$ and $1.2\%$. Within the multi-dimensional space of the surrogate model, 3D sub-spaces of primary variables, namely the dominant muscles and the global objective, flexion ratio, exhibit a path of optimal solutions. Within this optimal path, the muscle fibre pre-stretch of two flexors demonstrate a negative correlation, while, in contrast, the primary extensor, *m. triceps brachii* correlates positively to each of the flexors. Comparing the global optimum with four other designs along the optimal path, we saw large deviations, e.g., up to 15^∘^ in motion and up to 40% in muscle force. This underlines the importance of accurate determination of fibre pre-stretch in muscles, especially, their role in pathological muscular disorders and surgical applications such as free muscle or tendon transfer.

## Introduction

Impairments to the musculoskeletal system can lead to severe mobility restrictions. These often go hand-in-hand with a significant loss in quality of life. The design of new treatments or rehabilitation methods often lack effectiveness due to our limited understanding of the musculoskeletal system and its complex interactions. As with any complex system, it is often difficult, if not impossible to (experimentally) identify its system-inherent dependencies and interactions. Computational models are powerful tools to reveal and study the system behaviour.

As far as computational models of the musculoskeletal system are concerned, geometrical representations are typically obtained by segmenting anatomical images, e.g. (Fernandez et al. 2004), such as the ones provided by, e.g., the Visible Human Data set (Spitzer and Whitlock 1998) or medical imaging obtained via computed tomography (CT) scan, magnetic resonance imaging (MRI) or diffusion tensor imaging (DTI), e.g., Ramasamy et al. (2018). While such imaging data can provide highly detailed information on a system’s structure (anatomical modelling), it lacks information on functional aspects (physiological modelling). For example, MRI does not contain any information on the state of a muscle’s stretch or a muscle’s level of activation. Further, since almost all existing musculoskeletal system models are based on principles of multi-body dynamics, e.g., (Delp et al. 2007; Cleather and Bull 2015; Damsgaard et al. 2006; Rupp et al. 2015; Guo et al. 2019; Gfrerer and Simeon 2021), the anatomical data are often even further reduced to one-dimensional structures or point masses. In multi-body frameworks, for example, the anatomical representation of skeletal muscles is modelled as line of action pointing from origin to insertion. Its mechanical behaviour is described by Hill-type skeletal muscle elements (Hill 1938; Zajac 1989; Delp et al. 1990). Hence, the heterogeneous structural and functional complexity is reduced to a few lumped parameters. The reduced complexity leads to simulations that require a relative small amount of computational resources and run on desktop PCs.

Alternatives to multi-body simulation frameworks are three-dimensional, continuum–mechanical simulation frameworks modelling the skeletal muscles as volumetric objects, e.g., (Johansson et al. 2000; Zöllner et al. 2012; Lemos et al. 2007; Blemker et al. 2005; Chi et al. 2010; Röhrle and Pullan 2007; Röhrle et al. 2008; Yucesoy and Huijing 2012; Schmid et al. 2019). More recent works to this topic are, e.g., (Li et al. 2021; Elyasi et al. 2022; Zeng et al. 2023), where the active and passive behaviour of a single muscle has been studied without taken the antagonist muscles into consideration. Unlike Hill-type muscle models, continuum–mechanical modelling approaches are capable of taking into account a muscle’s spatial heterogeneity. The increase in spatial resolution and model complexity also comes with a substantial increase in computational effort, often restricting in silico studies to studies on single muscles and short contraction periods. For example, all above-mentioned continuum–mechanical models investigate the mechanical behaviour of skeletal muscles in isolation, none within a system’s setting. Muscles, however, do typically not act in isolation, but as agonist–antagonist muscle pair or functional muscle groups.

Continuum–mechanical frameworks of three-dimensional musculoskeletal systems are extremely rare. There exist only a very few: Fernandez and Hunter (2005) use inverse modelling to investigate the wrapping of muscles around the knee joint. Similar investigations for the use of multi-body systems were carried out by Guo et al. (2020). Lee et al. (2009) use a continuum–mechanical approach to provide "boundary conditions" for visualizing the motion of the skin. Wu et al. (2013) simulate facial expressions using a continuum–mechanical, multi-muscle framework. The muscles involved in facial expressions, however, do not exhibit the same agonist–antagonist muscle characteristics as required for joint motion. Röhrle et al. (2017) and Valentin et al. (2018) are so far the only ones that proposed forward simulations of agonist–antagonist muscle pairs, i.e. of the *m. biceps brachii* and *m. triceps brachii* as two upper limb muscles (Röhrle et al. 2017; Valentin et al. 2018), and demonstrated its feasibility. To the best knowledge of the authors, continuum–mechanical musculoskeletal system models consisting of more than two muscles do not currently exist.

While advanced imaging modalities can provide anatomical information (e.g. geometry), they typically lack information on its functional state, e.g. the current stretch (configuration) of the muscle and, hence, its reference configuration. This, however, is the key information for continuum–mechanical models and essential to achieve realistic motions of musculoskeletal systems. For studies on isolated muscles, this plays a minor role since those studies assume a freely prepared muscle as a starting point. The error introduced by assuming that the initial mesh is also the stress-free state is acceptable. Other parameters and state variables come with a larger uncertainty. This, however, does not hold for a musculoskeletal system.

The choice of stretch, in particular, the presence of a pre-stretch is directly linked to the force generating capabilities of the respective muscles. It directly influences the joint angle. Moreover, skeletal muscles are complex structures consisting of tendons, muscle tissue, and its transition zones. Each part has its own stiffness (pre-stretch) strongly influencing the overall behaviour of the system. Since the respective stretches cannot be directly determined from imaging data, we propose to employ a computational approach to determine realistic and heterogeneous (pre-)stretches. Persad et al. (2022) experimentally studied the passive tension and active muscle tension to external stimulation on patients with brachial plexus injury in an intraoperative study. They could emphasized the importance of muscle stretch of the transplanted muscle to stabilize and mobilize the imbalanced joint. We assume that the person-specific physiological range of joint motion can only be reached for appropriate (pre-)stretch values by considering flexion and extension motion with mutual dependency as the pre-stretch of the muscle fibres not only affects the active force generation, but also the development of passive stiffness in the musculoskeletal joint system during motion. Passive tissue stiffness, especially the stiffness of muscle fibres, leads to a nonlinear increase in resistance to limb movement when the fibres are passively stretched. In addition, the range of motion (RoM) changes as a result of musculoskeletal imbalance due to pathological or neuromuscular diseases (Scarr 2012; Chamberlain et al. 2013). This patient-specific situation of the muscle-joint system in the flexion and extension movement can also be taken into account by the targeted determination of fibre pre-stretch of each individual muscle. Hence, we use the resulting ratio of flexion and extension angles during joint motion as objective function to employ an optimization procedure. The objective is to fit the computed flexion ratio to the ratio which is measured during elbow motion with respect to the pre-stretches of individual muscle components. The proposed methodologies and hypothesis will be developed and tested using a novel five-muscle, continuum–mechanical, three-dimensional, musculoskeletal system model of the upper limb.

In short, (1) we provide for the first time a five-muscle, continuum–mechanical, three-dimensional musculoskeletal system model, (2) we carry out an optimization to determine the (pre-)stretches of skeletal muscle tissue that result in an optimal balanced joint motion in regard to the maximal flexion and extension joint motion of a healthy person, and (3) we demonstrated feasibility of forward simulations of a complex three-dimensional, continuum–mechanical, musculoskeletal system model.

## Models and methods

The geometrical representation of the upper limb model is based on the Visible Human dataset (Spitzer and Whitlock 1998) and discretized using the finite element (FE) method (Sect. 2.1). The mechanical description is based on the fundamentals of nonlinear continuum mechanics with a muscle-tendon-complex constitutive law similar to the one proposed by Röhrle et al. (2017) (Sects. 2.2 and 2.2.3). Appropriate boundary conditions and our novel approach to determine the (pre-)stretch of individual components based on a given anatomical model and the joint range of motion are outlined in Sect. 2.2.2 and Sect. 2.3, respectively.

### The anatomical upper limb model

**Fig. 1:**
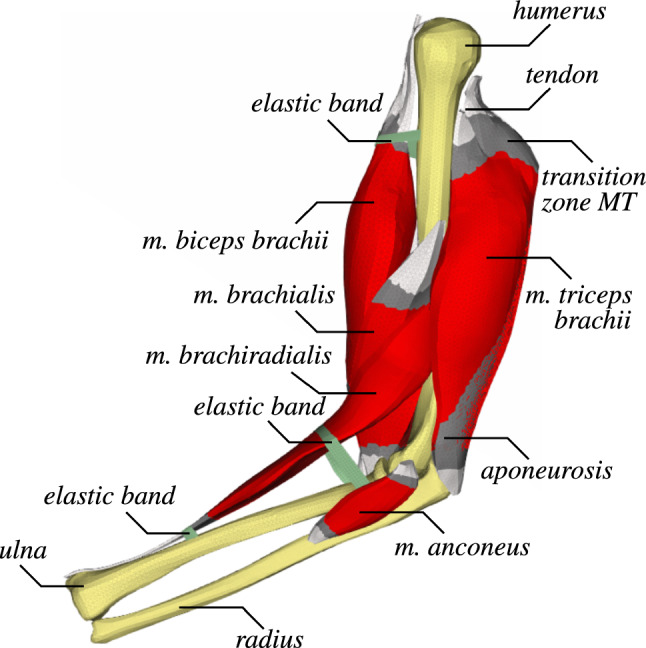
Lateral view on the FE-Model upper limb model: Muscle tissue is coloured in red, tendon in light grey, and the transition zone of muscle and tendon (muscle-tendon transition zones) in dark grey

Based on the Visible Human dataset (Spitzer and Whitlock 1998), we created CAD-models from the segmented major muscles of the upper limb, i.e. of the *m. biceps brachii*, *m. triceps brachii*, *m. brachialis*, *m. brachiradialis*, and *m. anconeus*. Meshing was achieved with the pre-processing tool ANSA version 19 (BETA CAE Systems SA International AG, CH). All volumetrical, deformable objects are meshed with tetrahedral elements consisting of elements with 4 nodes each. The final computational model of the upper limb is depicted in Fig. 1. The respective numbers of elements for the chosen discretisation of the respective parts are given in Table 1.

**Table 1:** Element number of the modelled muscles (including the tendons)

Muscle-Tendon part	*m. anconeus*	*m. biceps brachii*	*m. brachialis*	*m. brachiradialis*	*m. triceps brachii*
Tetrahedral elements (4-nodes)	52188	68292	184673	61271	272313

Note, Fig. 1 includes two non-anatomical elastic bands (in green) that have been included at the dorsal end of the *ulna*, close to the elbow joint and at the proximal end of the *humerus*. Based on the Reissner–Mindlin kinematic assumption, they have been modelled using a fully integrated 4-node shell element formulation with 1 mm thickness. These additional bands prevent the *m. brachiradialis* and *m. biceps brachii* to bend unnaturally. In reality, the *m. brachiradialis* and *m. biceps brachii*, for example, are covered by other muscles, deep fascia, which cover muscle tissue walls and separates individual muscles, retinaculum, which stabilizes mostly the tendons, and the fat-skin-layer. These structures prevent the respective muscles to bend unnaturally (upwards away from the joint/bone). If we would, like in reality, also consider all anatomical structures of the upper limb, the unnatural bending would not occur.

**Fig. 2:**
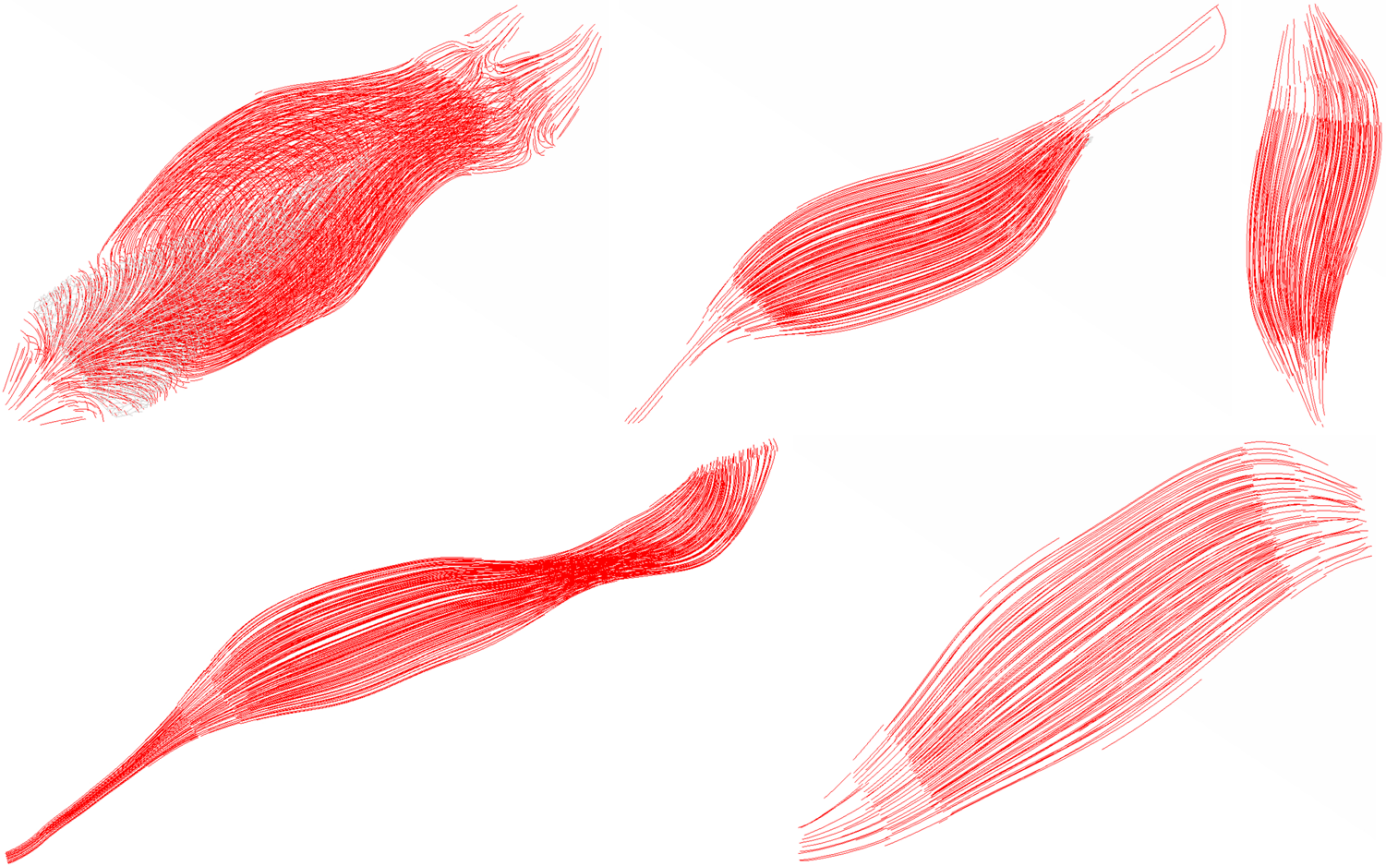
Muscle fibres from left to right are *m. triceps brachii*, *m. biceps brachii*, *m. brachialis*, *m. brachiradialis* and *m. anconeus*. Note, the individual figures are scaled to fit the overall figure height

Instead of extracting fibre orientations from the Visible Human data set, we employed a static thermal analysis generating a realistic fibre field (Handsfield et al. 2017). Hansfield showed that solving Laplacian-based equations, such as incompressible Stokes equations or the steady state 3D heat equations, could yield fibre orientations of gastrocnemius muscle with reasonable agreement with DTI datasets. In this method, the muscle-tendon-interfaces form the in- and outlets of the 3D-thermal flow. Note, if a muscle like the *m. triceps brachii* contains an aponeurosis, one has to account for the fact that fibres inserting into the tendon originate from different, here two, locations. This can be accounted for by defining appropriate boundary conditions (Choi and Blemker 2013). The results of the fibre orientations of the five arm muscles are compared with the illustrations from the standard textbook for general anatomy and musculoskeletal system (Schünke et al. 2018). From a visual point of view, the obtained fibre fields compare well, which correspond to the natural pathway of the fibres within the given muscle and tendon shape (Fig. 2).

### Constitutive modelling

The mechanical behaviour of the respective soft tissues is modelled using a three-dimensional, continuum–mechanical approach appealing to a macroscopic, phenomenological constitutive law. We follow the approach taken by Röhrle et al. (2017). It is assumed that the force developed by the muscle fibres can be additively split into an active and a passive part. Passive forces occur when fibres are stretched, while an active force is generated through a muscle contraction, i.e. the excitation-contraction pathway. The active part is also considered to be stretch-dependent. To take the stretch dependency into account, the active and passive contributions to the constitutive law are expressed in terms of the fibre’s length or stretch. The time-dependent viscoelastic behaviour of soft tissues is ignored. The same applies to the force-velocity relationship. Hence, we consider skeletal muscles, or more specifically, the muscle-tendon-complex, as hyperelastic, fibre-reinforced and quasi-incompressible material that can undergo large deformations. These assumptions are sufficient for the scope of the paper and coincide with the once for the theory of Finite Elasticity.

#### Govering equations

In the theory of Finite Elasticity, the deformed state of a body ${\mathcal {B}}$ is formulated in terms of the right or left Cauchy-Green tensor, i.e. $\textbf{C} = \textbf{F}^\textsf {T}_{}\textbf{F}$ or $\textbf{B} = \textbf{F}\textbf{F}^\textsf {T}_{}$ (with $\textbf{F}$ being the deformation gradient), respectively. Moreover, we assume slow movements such that we can neglect the acceleration terms. Thus, the balance of linear momentum reads

1 $$\begin{aligned} \begin{aligned}&\textsf {div}\,{\varvec{\sigma }}+\rho \textbf{g} = \textbf{0}, \end{aligned} \end{aligned}$$

where ${\varvec{\sigma }}$ is the Cauchy stress tensor, $\rho$ is the material density and $\textbf{g}$ denotes acceleration due to gravity. Further, with ${\varvec{\sigma }} = \textsf {det}\,\textbf{F}\ \textbf{F} \,\textbf{S} \,\textbf{F}^{\text{T}}$, Eq. (1) can be written in terms of the second Piola-Kirchhoff stress tensor, $\textbf{S}$. Further, we assume that the stress developed by the soft tissue can be additively split into an isotropic part that stems from the ground matrix, $\textbf{S}_\textsf {iso}^{}$, and into an anisotropic part that is associated with the fibre direction, $\textbf{S}_\textsf {aniso}^{}$. Likewise, we assume that the stress in fibre direction can be additively split into a passive, $\textbf{S}_\textsf {pas}^{}$, and an active contribution, $\textbf{S}_\textsf {act}$. The $\textbf{S}_\textsf {act}$ is hereby assumed to be the stress subject to maximal voluntary contraction. Hence, multiplying $\textbf{S}_\textsf {act}$ by a scalar associated with the level of active force production, i.e. the force generating capabilities of the respective fibre, $\alpha \ge 0$ (dropping the superscript *M* and subscript *n* for readability). Furthermore, we introduce a parameter $\gamma$, which is the volume fraction of skeletal muscle tissue at a material point. Then, we can express the second Piola-Kirchhoff stress tensor, $\textbf{S}$, as

2 $$\begin{aligned} \textbf{S} = \textbf{S}_\textsf {iso}^{} + \textbf{S}_\textsf {aniso}^{} = \textbf{S}_\textsf {iso}^{} + \textbf{S}_\textsf {pas}^{} + \gamma \, \alpha \, \textbf{S}_\textsf {act}. \end{aligned}$$

Note, by choosing $\gamma =0$, $\textbf{S}$ reduces to a stress tensor describing a purely passive material such as, for example, fat ($\textbf{S}_\textsf {pas}^{}\equiv \textbf{0}$) or tendons ($\textbf{S}_\textsf {pas}^{}\ne \textbf{0}$).

Following the strain energy formulation for the quasi-compressible isotropic soft tissue matrix outlined in (Crisfield 1997), the isotropic second Piola–Kirchhoff tensor reads

3 $$\begin{aligned} \textbf{S}_\textsf {iso}^{}= (B_1\textbf{I}+B_2\textbf{C}+B_3\textbf{C}^{-1})+k(\textsf {det}\,\textbf{F}-1)\,{I_3}^{1/2}\textbf{C}^{-1}, \end{aligned}$$

where *k* is the bulk modulus of the soft tissue matrix, and,

4 $$\begin{aligned} \begin{aligned} B_1&= 2C_1 I^{-1/3}_3 + 2C_2 I^{-2/3}_3 I_1, \\ B_2&= -2C_2 I^{-2/3}_3, \quad \text {and} \\ B_3&= -\nicefrac {2}{3}C_1 I^{-1/3}_3 I_1 - \nicefrac {4}{3}C_2 I^{-2/3}_3 I_2, \end{aligned} \end{aligned}$$

are the terms governed by invariants $I_1(\textbf{C}), I_2(\textbf{C})$ and $I_3(\textbf{C})$ of the right Cauchy-Green deformation tensor $\textbf{C}$. In Eq. 4, $C_1$ and $C_2$ are the Mooney-Rivlin material parameters. For material incompressibility, the bulk modulus *k* is adjusted with respect to the linear shear modulus $\mu =2(C_1+C_2)$ and the Possion ratio is assumed to be 0.4999. Further, the structural tensor $\textbf{M}$ is defined by the fibre direction with respect to the reference configuration, $\textbf{a}_0$, and is build by its dyadic product.

The active and passive contributions of fibres to the second Piola-Kirchhoff stress are defined as in (Röhrle et al. 2017) by

5 $$\begin{aligned} \begin{aligned} \textbf{S}_\textsf {pas}&= {\left\{ \begin{array}{ll} \dfrac{1}{\Lambda ^2_{\text{s}}}C_3^M\left( \Lambda ^{C_4^M}_{\text{s}}-1 \right) \textbf{M}, &{} \text {if } \Lambda _s\ge 1, \\ 0, &{} \text {otherwise}, \quad \textsf {and} \\ \end{array}\right. } \\ \textbf{S}_\textsf {act}&= {\left\{ \begin{array}{ll} \dfrac{\sigma _\textsf {max}}{\Lambda ^2_{\text{s}}} \exp {\Bigg (-\left| \dfrac{\nicefrac {\Lambda _s}{\Lambda _\textsf {opt}} - 1}{\Delta W_\textsf {asc}} \right| ^{\nu _\textsf {asc}}\Bigg )}\textbf{M}, \, \textsf {if } \Lambda _s\le \Lambda _\textsf {opt}, \\ \dfrac{\sigma _\textsf {max}}{\Lambda ^2_{\text{s}}} \exp \Bigg (-\left| \dfrac{\nicefrac {\Lambda _s}{\Lambda _\textsf {opt}} - 1}{\Delta W_\textsf {dsc}} \right| ^{\nu _\textsf {dsc}}\Bigg )\textbf{M}, \, \textsf {if } \Lambda _s>\Lambda _\textsf {opt}. \end{array}\right. } \end{aligned} \end{aligned}$$

Here $\Lambda_{\text{s}}$ is the fibre stretch, $\sigma _\textsf {max}$ is the potential Cauchy stress that a muscle can produce at its optimal length ($\Lambda _\textsf {opt}$) subject to a maximal voluntary contraction. It is assumed that the optimal fibre length, at which the muscle is capable of exhibiting its biggest force, is at $\Lambda _\textsf {opt}=1.3$.

The parameters $\Delta W_\text {asc}$ and $\nu _\textsf {asc}$ affect the ascending limb of the force-length relationship while $\Delta W_\text {dsc}$ and $\nu _\textsf {dsc}$ affect the stress behaviour at the descending limb of the force-length relationship. Hence, both effect the active fibre stress. Material parameters $C_3^M$ and $C_4^M$ describe the nonlinear exponential behaviour of the passive fibre stress. Each tissue, i.e. skeletal muscle and tendon tissue, is defined by its own material parameters. They are given in Sect. 2.2.3. We denote those for skeletal muscle tissue parameters with $C_3^M$ and $C_4^M$ and $C_3^T$ and $C_4^T$. The respective material parameters $C^{{MTZ}}_{{3}}$ and $C^{{MTZ}}_{{4}}$ define the material behaviour for the transition zone between muscle to tendon. They are obtained by linearly interpolating the tendon and skeletal muscle material parameters, i.e.

6 $$\begin{aligned} \begin{aligned} C^{{MTZ}}_{{3}}= \gamma \,C_3 + (1-\gamma )\,C_3^T \,, \\ C^{{MTZ}}_{{4}}= \gamma \,C_4 + (1-\gamma )\,C_4^T \,. \end{aligned} \end{aligned}$$

The above-described constitutive law was implemented as a user-defined subroutine in the commercial software package ANSYS LS-DYNA. The implementation of the constitutive law in LS-DYNA requires unlike most other software packages, the spatial counterpart of material tangent $\mathbb {C}$, which is denoted by $\mathbb {B}$. Tensor $\mathbb {C}$ is a fourth-order tensor obtained from the second-order Piola-Kirchhoff stress tensor by differentiating it with respect to $\textbf{C}$, i.e.

7 $$\begin{aligned} \mathbb {C} = \mathbb {C}_\textsf {mnop} = 2 \frac{\partial \textbf{S}}{\partial \textbf{C}}. \end{aligned}$$

Tensor $\varvec{\mathbb {B}}$ is obtained from $\mathbb {C}$ by a push-forward operation, i.e.

8 $$\begin{aligned} \mathbb {B} = (\textbf{F}\otimes \textbf{F})^{{\mathop {\textsf {T}}\limits ^{23}}}\mathbb {C}(\textbf{F}^{\textsf {T}}{\hspace{-1.66656pt}\otimes \hspace{1.66656pt}}\textbf{F}^\textsf {T})^{{\mathop {\textsf {T}}\limits ^{23}}}. \end{aligned}$$

Note, the superscript ^23^ above the transpose indicates that the transposition is defined by the exchange of second and third bases in the dyadic product. The artificially introduced bands are modelled using a Hookean constitutive law with a linear elastic material behaviour.

#### Contact and boundary conditions

In addition to find a geometrical representation of the musculoskeletal system, here, the upper limb, we need a well-defined functional description resulting in an uniquely solvable set of equations. This includes a set of balance equations, the respective constitutive laws, and coherent boundary and contact conditions.

**Fig. 3:**
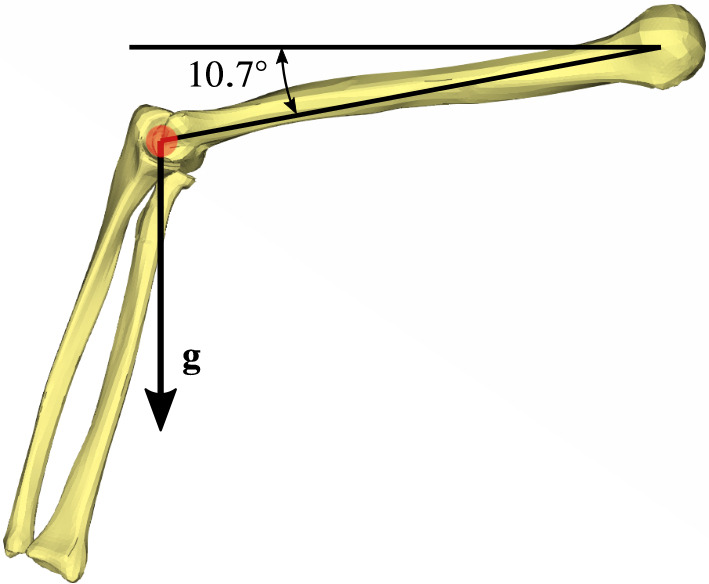
Angle of the elbow joint the position of the arm with the orientation of the gravitation force vector

We assume that the *humerus* is fixed in space. This restricts the arm movements to motions of the *ulna* and *radius*. Moreover, the elbow joint is assumed to be a hinge joint restricting the movement to one degree of freedom (dof), i.e. flexion and extension and ignoring supination, pronation, and the axial rotation of the lower arm. The rotation axis for flexion and extension is assumed to curve through the centre of the *epicondylus lateralis* and *medialis* of the *humerus*. Figure 3 depicts the centreline of the axis of rotation in red.

Flexion or extension is purely a result of varying the level of activation of a muscle with time, i.e. $\alpha ^M(t_n)=\alpha ^M_n$ for $n=1,\ldots ,N$, and muscle $M\in${*m. biceps brachii*, *m. triceps brachii*, *m. brachialis*, *m. brachiradialis*, *m. anconeus* }. Due to the change in activation, the respective exerted muscle forces change and the balance of forces determines the new elbow angle. This calculation is done for each time instance, hence, we consider the movement of the elbow as a quasi-static problem.

The *humerus* has been positioned in such a way that its gravitational force $\textbf{g}$ points straight downwards (normal to the ground). For other *humerus* positions, the agonists (flexor or extensor muscles) would produce an active muscle force against the gravitational force, while the antagonists would only have to be slightly activated to decelerate the motion (Latash 1998). Further, if we choose to let the gravitational force ${\textbf {g}}$ point upwards (${180}^{\circ }$ rotated), then the *m. triceps brachii* would not necessarily have to produce much active force in order to change the elbow angle. The elbow angle would be regulated with the help of gravitation. The entire elbow flexion and extension could be achieved by activating (or deactivating) the flexors. Hence, we chose herein the load case in which the flexor and extensor always have to work against gravitation in order to uplift the lower arm. The initial elbow angle, as depicted in Fig. 3, is a direct result from the anatomical model, i.e. the position the upper limb has been imaged and segmented.

Each considered muscle attaches to the *humerus* and *ulna* or *radius*. Since the *humerus* is fixed in space, we can assume that all proximal ends of the muscles (or tendons) are fixed in space as well. Fixing the respective muscle nodes attaching to the respective bones is done by assuming a tight contact between the respective parts and the *humerus*. The distal end of the muscles or tendons are in tight contact with the *ulna* or *radius*, respectively. Further, the inter-tissue contact, i.e. muscle-muscle or muscle-bone interaction including the tendons, is modelled using the well-known MORTAR-contact formulation (Puso and Laursen 2004).

#### Choice of material parameters

Our focus herein is to analyse the musculoskeletal system in general and not in a subject-specific manner. Hence, we choose herein a specific set of material parameters. Table 2 provides the material parameters for each component, i.e. muscle tissue, tendon tissue, and muscle-tendon transition zone. They are assumed to be equal for all muscles. Due to the phenomenological nature of the material description and the high inter-muscle material variability, it is nearly impossible to quantify an unique maximal muscle force value.

**Table 2:** Material parameter table of the arm model

Model parameter	Material parameter	Type	Value
Muscle	$C^{{M}}_{{1}}$	Isotropic	$7\times 10^{-3}$ MPa
	$C^{{M}}_{{2}}$	Isotropic	$7\times 10^{-4}$ MPa
	*k*	Isotropic	7.2 MPa
	$C^{{M}}_{{3}}$	Fibre (passive)	$2\times 10^{-5}$ MPa
	$C^{{M}}_{{4}}$	Fibre (passive)	21 (−)
	$\sigma _\textsf {max}$	Fibre (active)	0.3 MPa
	$\Delta W_\textsf {asc}$	Fibre (active)	0.15 (−)
	$\Delta W_\textsf {dsc}$	Fibre (active)	0.16 (−)
	$\nu _\textsf {asc}$	Fibre (active)	2 (−)
	$\nu _\textsf {dsc}$	Fibre (active)	6 (−)
	$\Lambda _\textsf {opt}$	Fibre (active)	1.3 (−)
Tendon	$C^{{T}}_{{1}}$	Isotropic	$7\times 10^{-2}$ MPa
	$C^{{T}}_{{2}}$	Isotropic	$7\times 10^{-3}$ MPa
	$C^{{T}}_{{3}}$	Fibre (passive)	${5}\times 10^{-1}$ MPa
	$C^{{T}}_{{4}}$	Fibre (passive)	25 (−)
Transition zone	$C^{{MTZ}}_{{1}}$	Isotropic	$7\times 10^{-2}$ MPa
	$C^{{MTZ}}_{{2}}$	Isotropic	$7\times 10^{-3}$ MPa
	$\gamma$	Fibre (passive)	$4\times 10^{-2}$ (−)
Elastic band	*E*	Young’s modulus	$1\times 10^{2}$ MPa
	$\nu$	Poisson number	${3}\times 10^{-1}$ (−)

The bony structures, i.e. *ulna*, *radius* and *humerus*, are assumed to be rigid bodies. To setup the contact problem, however, one requires all bodies, which are in contact, to be elastic. Hence, we define bones as a linear elastic material with a Young’s modulus of $1100\,{\text {MPa}}$ and a Poisson’s ratio of 0.3.

#### Pre-, initial-, and fibre-stretch

In any configuration, i.e. arm position, the soft tissues may be in a stretched state (including a muscles pre-stretch) and may exhibit nonzero stresses (which depend on the stretched state). This needs to be considered. We define the actual fibre stretch, $\Lambda_{\textsf{s}}$, as

9 $$\begin{aligned} \begin{aligned} \varvec\Lambda ^{{}}_{\mathrm{s}} =\bar{ \varvec\Lambda }^{{}}_{\mathrm{s}}(\Theta ^{{}}_{\mathrm{ini}}) +\varvec \Lambda ^{{}}_{\mathrm{C}} = \varvec\Lambda ^{{}}_{\mathrm{ini}} + \Delta \varvec\Lambda ^{{}}_{\mathrm{C}} + \varvec\Lambda ^{{}}_{\mathrm{C}}, \end{aligned} \end{aligned}$$

where ${\varvec\Lambda }^{{}}_{ {\mathrm{ini}}}$ is a spatially constant initial value that we assume a priori for the overall tissue. Its value can vary between the minimal and maximal stretch of the tissue. Further, this value is used to optimize for $\bar{\varvec\Lambda }^{{}}_{\mathrm {s}}$ in such a way that the modelled musculoskeletal system can recast the respective joint range of motion. Note, $\bar{\varvec\Lambda }^{{}}_{\mathrm{s}}$ depends on the initial joint angle, $\Theta ^{{}}_{\mathrm{ini}}$, of the model as it was segmented and generated from imaging modalities. Further, $\Delta \varvec\Lambda ^{{}}_{\mathrm{{C}}}$ is computed during the pre-stretch initialization process and represents additional muscle fibre stretches caused by passive arm motions. These are on the one hand the joint rotation caused by applying the initial fibre pre-stretches and on the other hand the back rotation of the joint from the new steady-state joint position back to the initial configuration $\Theta ^{{}}_{\text{{ini}}}$. Further passive or active extensions or contractions of the muscle fibres during the forward analysis of the joint system are represented by $\varvec\Lambda ^{{}}_{\mathrm{{C}}}$, while the initialized muscle’s pre-stretch for the reference configuration $\bar{\varvec{\Lambda }}^{{}}_{\mathrm{{s}}}$ is kept constant. The index *C* indicates that $\varvec\Lambda ^{{}}_{\mathrm{{C}}}$ is computed at each material point of the respective soft tissue using the right Cauchy-Green strain tensor $\textbf{C}$, i.e. by

10 $$\begin{aligned} \begin{aligned} \varvec\Lambda _{\text{c}}^2:= I_4(\textbf{C}, \textbf{M})&= \textbf{M}: \textbf{C},&\text {with} \quad \textbf{M}&= \textbf{a}_0{\otimes \hspace{1.66656pt}}\textbf{a}_0. \end{aligned} \end{aligned}$$

Likewise, $\Delta \varvec\Lambda ^{{}}_{\mathrm{\textbf{C}}}$, which exists due to the initialization of pre-stretches, is also determined by Eq. (10). In other words, we do not determine the muscle fibre pre-stretch directly, only indirectly by adjusting the overall ${\textbf{S}}$ in such a way that it mimics the actual motion. Once we obtained the right spatial distribution of the fibre pre-stretches, $\bar{\varvec{\Lambda }}_{{\text{s}}}$, we keep them fixed for our forward simulations.

### Optimization strategy for computing the pre-stretch of the individual muscle-tendon-complex components

In Eq. (9), we introduced the muscle fibre pre-stretch. The intrinsic pre-stretches of the individual muscles are directly linked to the joint angle—independent weather the muscles are in an active or passive state. To define the pre-stretch for a particular state of the musculoskeletal system, or the fibre stretch that intrinsically considers the muscle’s pre-stretch, we first define the arm position for which the muscle’s pre-stretch will be determined. We refer to this position as the joint angle position in which no gravity is acting (cf. Step LS-2 in Sect. 2.3.2). Applying gravity to the system causes some arm motion. This motion varies with the pre-stretch acting on the muscle system, because each pre-stretch induces a specific distribution of stiffness at the joints (passive joint resistance, cf. Step LS-3 in Sect. 2.3.2). The respective motions of the arm result from muscle contractions and are directly related to that individual arm position (cf. Step LS-4 in Sect. 2.3.2 and subsequent load cases). Consequently, the optimization procedure of the muscle’s fibre pre-stretch for the initial arm position considers several forces acting on the system’s balance and directly influences the active arm motion. These are, e.g. the passive tissue deformations due to the tissues’ pre-stretch, gravity, the passive muscle stretch induced by the arm position (the antagonist serves as a resistance to the agonist), or additional structures or mechanism providing joint stiffness.

The generation of muscle force is always linked to the contraction behaviour of the muscle, which follows the well known force-length curve. In case the pre-stretch is less than the optimal length, the active force generation follows downwards the ascending part of the active force-length curve due the fibre contraction (shortening of the fibres), otherwise it follows the descending part upwards. Too high or too low pre-stretch values could be very inefficient for generating active arm motions. This means that, depending on the initially defined arm position, the muscle’s pre-stretch must be balanced in such a way that the joint works equally efficient during flexion and extension. This natural synergistic interplay of muscles between passive joint stiffness (resistance) and active work is very efficient for the joint’s physiological range of motion, but can become very inefficient if the wrong pre-stretches are assumed.

#### Description of the lower arm kinematics

**Fig. 4:**
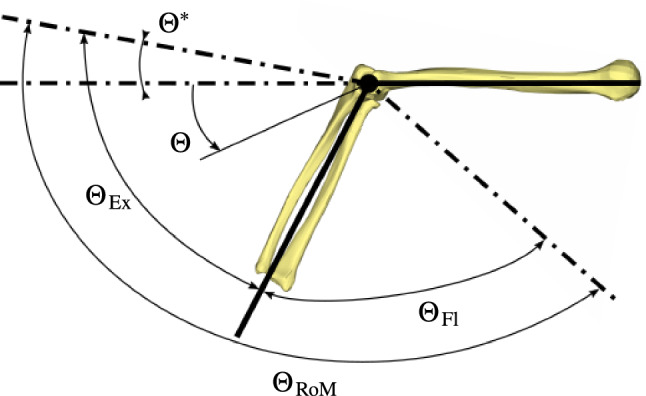
Illustration of the lower arm rotation angles

Within this work, we consider the rotation of the lower arm, i.e. the rotation between the *humerus* and the *ulna* and *radius*, cf. Fig. 4. The relative motion between the *ulna* and *radius* is not considered. The origin of rotation angle $\Theta$ is the neutral zero position of the stretched lower arm with respect to the straight line of the *humerus*. Proceeding from the neutral-zero position $\Theta = 0^\circ$, the lower arm can be overstretch in extension by a maximum of ${\Theta }^{*}$ degrees. Based on the initial position of lower arm $\Theta _{\mathrm{\text{ini}}}$, it maximally rotates in extension by ${\Theta }_{\mathrm{\text{Ex}}}$ degrees and by ${\Theta }_{\mathrm{\text{Fl}}}$ degrees in flexion. The sum of both rotations defines the total range of motion (RoM) and is given by ${\Theta }_{\mathrm{\text{RoM}}}$.

Table 3 provides for the upper arm model, as introduced in Sect. 2.1, the experimentally measured flexion-extension elbow joint rotation angles $\widetilde{\Theta }$ of a healthy person (Schünke et al. 2018). The data are with respect to the initial arm position of the finite element model, i.e. with respect to $\Theta _{\mathrm{\text{ini}}}:={{\Theta = 62.1^\circ}}$ (cf. Fig. 4).

**Table 3:** Arm rotation angles of a healthy person (a tilde denotes an experimentally measured quantity)

Measured angles	
$\widetilde{\Theta }^{*}$	= $-10^\circ$
$\widetilde{\Theta }_{\text{RoM}}$	= $150^\circ$
$\widetilde{\Theta }_{\text{Ex}}$	= $72.1^\circ$
$\widetilde{\Theta }_{\mathrm{\text{Fl}}}$	= $77.9^\circ$
$\Theta _{\mathrm{\text{ini}}}$	= $62.1^\circ$

The right balance of the muscle’s pre-stretch with respect to flexion and extension is essential for predicting realistic muscle forces. The stretch of a muscle directly influences the motion in each direction. Thus, the current arm position plays a major role in determining a muscle’s pre-stretch.

Depending on arm position $\Theta$, a ratio between flexion and extension gives a good indication how much rotation is possible relatively to extension or flexion. Hence, we introduce a flexion ratio *r* that directly depends on the achievable arm rotation angle, $\Theta _{\mathrm{\text{ini}}}$, i.e.

11 $$\begin{aligned} \begin{aligned} r(\textbf{s}; \Theta _{\mathrm{ \text{ini}}}) = \frac{\Theta _{\,\mathrm {\text{Fl}}}(\textbf{s};{ \Theta _{\mathrm {\text{ini}}})}}{\Theta _{\,\mathrm {\text{RoM}}}(\textbf{s}; \Theta _{\mathrm {\text{ ini}}})} , \end{aligned} \end{aligned}$$

where $\textbf{s}$ denotes a design point (DP) providing in vector notation the muscle pre-stretches of the musculoskeletal system (the set of design points is later used for a sensitivity study, cf. Sect. 3.1.1).

12 $$\begin{aligned} \begin{aligned} \Theta _{\mathrm {\text{RoM}}}(\textbf{s};{ \Theta _{\,\mathrm {\text{ini}}})}&=\Theta _{\,\mathrm{\text{Fl}}}(\textbf{s};{ \Theta _{\,\mathrm {\text{ini}})}}+\Theta _{\,\mathrm {\text{Ex}}}(\textbf{s};{ \Theta _{\,\mathrm {\text{ini}}})}\, \quad \text {with} \\ \Theta _{\,\mathrm {\text{Ex}}}(\textbf{s};{ \Theta _{\,\mathrm{\text{ini}}})}&= \Theta _{\,\mathrm{\text{ini}}} - \Theta (\textbf{s})\, \\\Theta _{\,\mathrm{\text{Fl}}}(\textbf{s};{ \Theta _{\,\mathrm{\text{ini}}})}&= \Theta (\textbf{s}) - \Theta _{\,\mathrm{\text{ini}}}\,. \end{aligned} \end{aligned}$$

Therein, $\Theta _{\,\mathrm{\text{Fl}}}$ and $\Theta _{\,\mathrm{\text{Ex}}}$ represent the maximally possible flexion and extension angles starting from $\Theta _{\,\mathrm{\text{ini}}}$, i.e., respectively, contracting all flexors and extensors maximally, and the initial idle state position of the arm.

This parameter serves also as the target value for an optimization procedure to determine the respective pre-stretches. The maximal physiological motion is not only limited due to the force producing capabilities of the muscles but also mechanically by the olecranon, i.e. the posterior process of the ulna. This limitation leads to a restriction for the measured range of motion of $\widetilde{\Theta }_{\text{RoM}}=150^\circ$ for general healthy human. Therewith, from Eq. (11), the parameters in Table 3, and the initial arm position, the measurable flexion ratio, ${\widetilde{r}}$, of a healthy person yields ${\widetilde{r}}(\Theta )\big |_{\theta =62.1^\circ }=0.519.$ Note, for the ${\widetilde{r}}$, which is based on experimental data, the argument $\textbf{s}$ is omitted, since the pre-stretches are inherently given.

Based on an experimentally determined RoM and initial arm position, the corresponding values of Table 3 and ${\widetilde{r}}$ can be determined in a subject-specific way. This even holds for pathological cases. While the RoM is measured using motion capture or any other means, the initial arm position can be determined directly from the underlying medical images, e.g., obtained by means of MRI.

#### Optimization problem

To obtain realistic muscle pre-stretches, we propose a computational approach. We assume that the best joint range of motion is achieved, if the pre-stretches are, from a systems point of view, "optimal" balanced to the flexion and extension motion in respect to the initial arm position. The optimal balanced muscle pre-stretch will deliver a more force efficient arm rotation in both directions. For setting up the respective optimization problem, we propose to use the ratio between maximal flexion and maximal extension, i.e. flexion ratio ${\widetilde{r}}$, as an optimization criterion, which is related to the reached RoM. The rotation angles are taken from the FE analysis received for the activation level $\alpha =1$ for all active flexors or all active extensors while the antagonist muscles are inactive, respectively. Then, the resulting optimization problem for determining the respective pre-stretches $\textbf{s}$, i.e. the pre-stretches inherent to the five individual muscles, is given by

13 $$\begin{aligned} \begin{aligned} \underset{\mathrm{\textbf{s} \in \textbf{D}}}{\text {argmin}} \, {F}(\textbf{s};\Theta _{\,\mathrm{ini}} )= \underset{\mathrm{\textbf{s} \in \textbf{D}}}{\text {argmin}} \,\Big \Vert r^{\mathrm{sim}}(\textbf{s}; \Theta _{\,\mathrm{ini}})-{\tilde{r}}(\Theta _{\,\mathrm{ini})} \Big \Vert , \end{aligned} \end{aligned}$$

where *F* denotes the objective function and $r^{\text{sim}}$ is the flexion ratio based on the respective FE simulation. The parameter $\textbf{s}$ denotes one point of the overall set of design points $\textbf{D}$. We have used for each muscle pre-stretch the equal design space, which are given in (Table 5). The result of the optimization problem in Eq. (13) is the set of optimal initial muscles’ pre-stretch values, $\textbf{s}^{\mathrm{opt}}_{{}}$. Note, as we always consider within this work the same $\Theta _{\,\mathrm{ini}}$= $62.1^\circ$, we will omit, for the sake of readability, this variable from now onwards. In total, $\textbf{s}$ is in our five-muscle upper arm model a fifteen-dimensional vector consisting of the pre-stretches of the muscle, the tendon and the transition zone component of each of the five muscles.

To simplify the optimization tasks, the fibre pre-stretch parameters for the tendons and muscle-tendon transition zones are pre-defined for all muscles and are not part of the optimisation procedure. This choice was made based on a pre-analysis of the system behaviour for all five muscles. This pre-analysis exhibited that the pre-stretches for the tendons and muscle-tendon transition zones only minimally influence the RoM. In contrast to this, the muscle’s pre-stretch is directly and strongly correlated with the level of activation. The pre-determined (and assumed) pre-stretch values for the tendons and the muscle-tendon transition zones are given in Table 4.

**Table 4:** Initial fibre pre-stretches for the tendons and the muscle-tendon transition zones

Muscle	*m. anconeus*	*m. biceps brachii*	*m. brachialis*	*m. brachiradialis*	*m. triceps brachii*
Initial tendon fibre pre-stretches	0.00675	0.00805	0.00145	0.00168	0.0095
Initial transition zones fibre pre-stretches	0.01161	0.03005	0.00822	0.01674	0.0146

Before computing $r^{\mathrm{sim}}$, we first note that the choice of constitutive model (Sect. 2.2) is derived from a potential and it includes no energy dissipation such as viscosity, plasticity or friction during contact. This in turn means that the order of muscle activation does not play a role in calculating the final rotation. Furthermore, we note that the role of the five muscles, which result in a movement of the *ulna* and *radius* with respect to the *humerus*, is well known. The *m. biceps brachii*, *m. brachiradialis*, and *m. brachialis* are considered to be flexors, *m. triceps brachii* and *m. anconeus* are considered as the extensors.

The optimization problem is solved in LS-OPT by SQP (Sequential Quadratic Programming). For general references on the SQP, the interested reader is referred to relevant well-known books and papers of this topic, e.g. (Boggs and Tolle 1995; Bertsekas 1997; Luenberger and Ye 2015) or the manual of LS-OPT.^1^ The evaluation of the optimization function requires the computation of $r^{\mathrm{sim}}$ for each design point (DP) is achieved by executing the following load steps (LS):

1. After setting for each muscle the respective fibre pre-stretch, $\Lambda ^{\mathrm{i}}_{\mathrm{ini}}$ with $\textbf{s}=(\Lambda ^{{}}_{\mathrm{ini}})_{i=1,\ldots ,K}$ and *K* being the number of muscles, we compute the system’s static equilibrium (steady state). Depending on the choice of pre-stretch, this will result in a change of configuration.
2. We apply a joint moment to the elbow such that the bones will be rotated back to the original configuration. The joint moment is found by one parameter optimization or, if needed, by bisection.
3. We apply the gravitational force to the system.
4. We fully activate all extensor muscles, i.e. $\alpha ^{{i}}_{{}}=1$ for $i \in \{\textit{m.~triceps brachii}, \textit{m.~anconeus} \}$ and 0 all the others. Overall, we denote that level of activation with $\varvec{\alpha }^{{}}_{\mathrm {Ex}}=[ 0, 0, 0, 1, 1 ]^T$. The resulting elbow joint angle is denoted with $\Theta _{\,\mathrm{Ex}}$ .
5. We deactivate (${\varvec{\alpha }}=$ **0)** all extensor muscles.
6. We fully activate all flexor muscle, i.e. $\alpha ^{{i}}_{{}}=1$ for $i \in \{\textit{m.~biceps brachii}, \textit{m.~brachiradialis}, \textit{m.~brachialis} \}$ and 0 all the others. The resulting elbow joint angle is denoted with $\Theta _{\,\mathrm{Fl}}$ and the level of activation ${\varvec{\alpha }}^{{}}_{\mathrm{Fl}}=[ 1, 1, 1, 0, 0 ]^T$.
7. We compute the flexion ratio $r^{\mathrm{sim}}$ based on Eqs. (11) and (12).

### Surrogate modelling, accuracy estimates, and parameter sensitivity indication

To reduce computational time for optimizing the pre-stretch of the muscles, i.e. minimizing Eq. (13), or as in this case to make it feasible at all, a surrogate model strategy is applied. Surrogate modelling or metamodelling approaches are well-established and well-suited methods for efficient investigation of complex and computationally demanding models. Utilizing this class of methods for our context, we seek to derive as metamodel a surrogate approximating the respective objective function $F(\textbf{s})$, i.e. the flexion ration $r^{\mathrm {sim}}(\textbf{s})$. To provide sufficient accuracy to the metamodel by recovering the design space as best as possible, here we used the well-known space-filling approach as provided by LS-OPT with default settings. In the current study, the metamodel is constructed using radial basis function networks (RBF), with Hardy’s multi-quadrics (Hardy 1990) employed as the basis function. A detailed description of methods is given in the manual LS-OPT (Stander et al. 2015). The defined design space of the muscle fibre pre-stretches $\textbf{s}$ is given in Table 5. This choice of the design space is broad because we did not want to make strict assumptions on the approximation field of the fibre pre-stretches.

**Table 5:** Design space of the initial muscle fibre stretch for the sampling of design points

Muscle	*m. anconeus*	*m. biceps brachii*	*m. brachialis*	*m. brachiradialis*	*m. triceps brachii*
Initial fibre pre-stretch	$0.05\le \Lambda ^{an}_{\mathrm{ ini}} \le 0.45$	$0.05\le \Lambda ^{bi}_{\mathrm{ ini}} \le 0.45$	$0.05\le \Lambda ^{ba}_{\mathrm{ ini}} \le 0.45$	$0.05\le \Lambda ^{br}_{\mathrm{ ini}} \le 0.45$	$0.05\le \Lambda ^{tr}_{\mathrm{ ini}} \le 0.45$

To obtain some insights into model accuracy, we use the general root-mean-square (RMS) error measure,

14 $$\begin{aligned} \begin{aligned} \epsilon _{{RMS}}&= \sqrt{\frac{1}{N}\sum ^N_{i=1}(r_i-{\hat{r}}_i)^2}\, , \end{aligned} \end{aligned}$$

where $r_i$ is the calculated flexion ratio obtained from the FE analysis and ${\hat{r}}_i$ reflects the respective one predicted by the surrogate model. The summation index, *i*, refers to a DP and *N* is the total number of DPs. In addition, we consider the accuracy measurement coefficient $R^2$, which is defined as

15 $$\begin{aligned} \begin{aligned} R^2&= \frac{\sum ^N_{i=1}({\hat{r}}_i-\bar{r}_i)^2}{\sum ^N_{i=1}(r_i-\bar{r}_i)^2}. \end{aligned} \end{aligned}$$

It defines the capacity of the surrogate model to identify the variances of the design response. Therein, $\bar{r}_i$ is the mean response, ${\hat{r}}_i$ is the flexion ratio predicted by the surrogate model, and $r_i$ the respective value obtained by means of the full finite element simulation. Like before, *i* refers to a DP and *N* to the total number of DPs.

Further, a Global Sensitivity Analysis (GSA) (Sobol’s method, based on ANOVA, (Sobol 2001)) is employed to predict the influence of a particular variable on the outcome. As the Global Sensitivity analysis is based on absolute and normalized values, the influence of a parameter on multiple responses, e.g. on the full load case or the entire optimization, can be obtained by summing up the individual values.

## Results

First, we report in Chapter 3.1 on the impact of a muscle fibre pre-stretch on the overall behaviour of the musculoskeletal system. This includes the impact of the surrogate model’s accuracy and its sensitivity on the overall system response. Once an optimal pre-stretch has been determined, we highlight in Chapter 3.2 the models ability to reproduce realistic motions and muscle forces based on prescribed activation patterns based on heterogeneous spatially-dependent muscle fibre pre-stretches.

### Analysis of the muscle’s pre-stretch with respect to arm motion

First, we present the results of the surrogate-model-based sensitivity analysis of the defined response functions and the objective function with respect to the muscle’s pre-stretch. Additionally, results on the accuracy of the approximation are presented. For the three-dimensional visualization, the surrogate’s model regression surfaces are utilised to investigate the pre-stretch’s influence on the objective function/range of motion, which is essential for assessing model quality. After establishing the surrogate model’s validity and accuracy estimates, the results of the pre-stretch optimization are presented. Given the large amount of data and DPs, we conduct a detailed parameter study of the pre-stretch behaviour to the arm motion by comparing the solution of the optimal design (OD) with the solution at selected DPs. For this purpose, we selected DPs close to the optimum to investigate changes to the arm’s kinematics due to changes/variations in the pre-stretch. The calculation time of the implicit analysis varies with every individual design point. It amounts between 5-6 h for LS-1 to LS-6 using 4 CPUs, while LS-7 is a post-processing step. We used a Symmetric Multi-Processing system (SMP) for the calculations.

#### Sensitivity investigation

An accurate metamodel requires a well approximated design space with sufficient experimental design points (DPs). Although LS-OPT suggests for 5 design variables 32 DPs, we stayed conservative and selected instead 150 DPs. This choice was made to also ensure high accuracy for highly nonlinear responses.

**Fig. 5:**
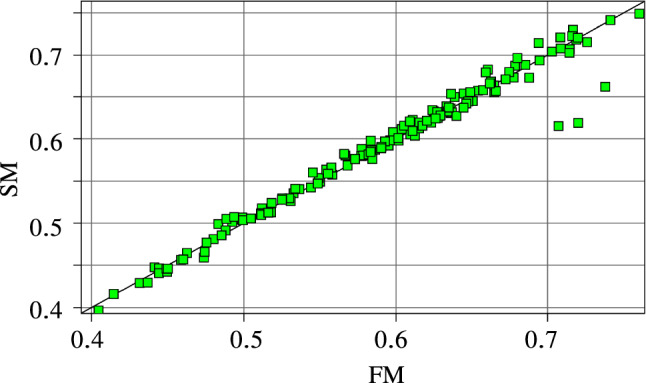
Surrogate model accuracy for the flexion ratio *r*. FM are the computed values using the full model and SM the predicted values of the metamodel

First, we verify the surrogate model’s accuracy. Figure 5 compares for different pre-stretches, i.e. the selected DPs, the values computed by the finite element model (FM) of the flexion ratio *r* in Eq. (13) with the values based on the surrogate model (SM), i.e., ${\hat{r}}$.

The black line with slope 1 denotes $100\%$ accuracy between the computed and predicted values, i.e. $R^2=1$ and $\epsilon _{\textit{RMS}}=0$. Determining for each DP the ratio between the computed and predicted value and, then, computing based on this series the coefficient of determination, $R^2$ and the root mean square, $\epsilon _{\textit{RMS}}$, we obtain 0.956 and $2.97\%$, respectively. Overall, there were only three outliers. They were associated with large flexion ratios, which are not likely to occur.

**Fig. 6:**
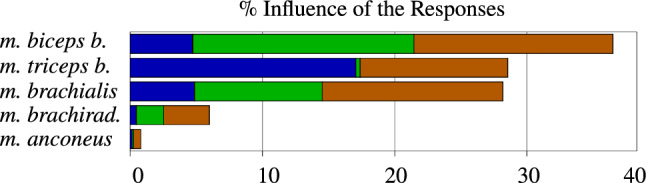
GSA of the muscle fibre pre-stretch to Extension ($\Theta _{\text{Ex}}$) (blue), Flexion ($\Theta _{\text{Fl}}$) (green) and flexion ratio (*r*) (brown)

To perform a GSA study, we evaluated a total of 20 000 integration points on the metamodel chosen by a Monte-Carlo approach. The result of the nonlinear GSA is depicted in Fig. 6. It clearly shows, as it was be expected, that the muscle fibre pre-stretches of the *m. biceps brachii* (flexor), *m. triceps brachii* (extensor), and *m. brachialis* (flexor) impact the flexion ratio (brown bar) most and, hence, dominate the objective function and, therefore, the overall range of motion.

In total, a change in pre-stretch, i.e. initial stretch, in these three muscles significantly influences the considered elbow motion, i.e. range of motion. They account for 91%, while the *m. brachiradialis* and *m. anconeus* only account for $7.8\,\%$ and $1.2\,\%$, respectively. As far as $\Theta _{\,\mathrm{Fl}}$ (LS-6) is concerned, the influence of the pre-stretch of the three most dominant muscles amounts again to $92.4\,\%$. Only considering the flexors (without *m. brachiradialis*, which accounts for $7.1\,\%$) results for $\Theta _{\,\mathrm{Fl}}$ in $91,3\,\%$. For extension (LS-4), $\Theta _{\,\text{Ex}}$ exhibits for these three muscles an even stronger but similar behaviour (amounting to $97,8\,\%$). The influence of the extensor’s pre-stretch, i.e., *m. triceps brachii* and *m. anconeus*, on $\Theta _{\,\mathrm{Fl}}$ ($1.5\,\%$) is remarkable low. In contrary, the flexors’ pre-stretch strongly effects the motion ($36.8\,\%$). This is due to the passive muscle fibre stiffness, which gives a resistance depending on the amount of the muscle’s pre-stretches. The GSA provides us with guidance and allows us to mainly focus our investigations on the three main muscles, i.e., *m. biceps brachii*, *m. triceps brachii*, and *m. brachialis*.

#### Surrogate-based pre-stretch optimization

**Fig. 7:**
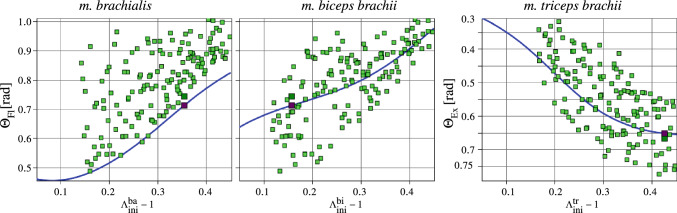
2D regression curves of the response variables flexion and extension rotation (blue line) in regard to the muscle fibre pre-stretches. The computed optimum is the dark green design point and the predicted optimum is the brown design point. (left) and (middle) is the arm flexion and (right) extension

To visualize multi-dimensional functional responses, we take a step-by-step approach. We first focus on individual muscles, i.e. the *m. biceps brachii* and *m. brachialis* as flexors and the *m. triceps brachii* as sole extensor—the prominante ones of the GSA (Fig. 6). Figure 7 depicts 2D regression curves of the arm flexion and extension response functions for the *m. brachialis*, *m. biceps brachii*, and *m. triceps brachii* with respect to its muscle fibre pre-stretch parameter $\Lambda ^{{M}}_{\text{ini}}$, $M\in \{\textit{m.~brachialis}, \textit{m.~biceps brachii}, \textit{m.~triceps brachii} \}$. For each 2D regression curve of an illustrated pre-stretch parameter all remaining parameters are constant at the value of the OD. The optimized initial fibre stretch parameters, $\Lambda ^{{}}_{\mathrm{ini}}$, of the OD are given in Table 6. One could be observed that the DPs are coming closer together (clustering) with increase in the fibre stretch. This behaviour is obvious because of the high fibre pre-stretch values, which are effecting more the passive joint stiffness (resistance–antagonist) and also generating more active muscle forces (motion–agonist).

**Fig. 8:**
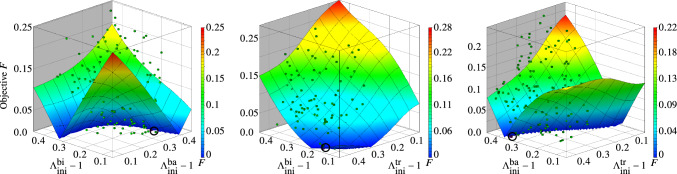
Metamodels of the objective function *F* related to each two dominant muscle fibre pre-stretch variables, respectively: (left) *m. biceps brachii* versus *m. brachialis*, (middle) *m. biceps brachii* versus *m. triceps brachii* and (right) *m. brachialis* versus *m. triceps brachii*

**Fig. 9:**
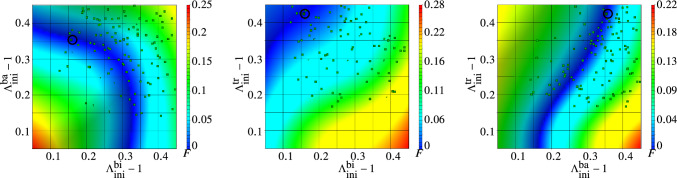
Top view of the metamodels of the objective function *F* related to each two dominant muscles, respectively: (left) *m. biceps brachii* versus *m. brachialis*, (middle) *m. biceps brachii* versus *m. triceps brachii* and (right) *m. brachialis* versus *m. triceps brachii*

Figures 8 and 9 show two alternative visualizations of the response surfaces with respect to two effective design variables (left: the initial stretch relationships for *m. biceps brachii* and *m. brachialis*, middle: for *m. biceps brachii* and *m. triceps brachii*, right: *m. brachialis* and *m. triceps brachii*). While Fig. 8 provides a three-dimensional iso-metric view on the predicted objective function, Fig. 9 provides a two-dimensional projection onto the design parameter space (top view). All figures show a distinct path of possible optimal solutions on the metamodels for which the objective function is nearly zero.

Following the dark blue path of possible optimal solutions for the objective function (near zero) shown in Fig. 9 (left), it infers that the initial pres-stretches of *m. brachialis* up to 0.2 is less relevant for optimal solutions, while *m. biceps brachii* is dominating the solution path with about 0.3. The initial pre-stretches of *m. brachialis* become more effective for values higher than 0.2 with the consequences that the values for *m. biceps brachii* are decreasing. Figure 9 (middle) informs that the objective function favours optimal solutions with low initial fibre stretch values of *m. biceps brachii* and high values of *m. triceps brachii*, whereas the values for *m. brachialis* are in a similar range with a light trend to a higher values for *m. triceps brachii* (right). This corresponds to a general expectation that the relationship of the initial pre-stretches of *m. biceps brachii* and *m. brachialis* is negatively correlated in regard to the optimal solution path, i.e. an increase in one parameter leads to decrease of the other to fulfill the optimization criterion, while we found a positive correlation to *m. triceps brachii*. In this case an increase of the initial pres-stretch of the flexors along the optimal solution path leads to an increase in values for *m. triceps brachii* too. One needs to keep in mind that this is only one way for visualizing the multi-dimensional objective function in several three-dimensional illustrations. Therewith, the dependency of one parameter (the initial pre-stretch of one muscle) on the objective function with respect to another one could be studied. The individual metamodels, which are a sub-domain of the overall solution of the complete surrogate model, makes possible to better understand the complexity of the overall system.

The optimal design (OD) is found on the surrogate model and is visualized in Figs. 8 and 9 by colouring the design point in dark and highlighting it with a black circle for better identification. The objective function value for OD, as given in Table 6, is ${F^{{}}_{\text{SM}}(\text{OD})=1.075e-9}$. The FE model for OD results in ${F^{{}}_{\text{FM}}(\text{OD})=0.008457}$. Generally as default termination an objective function tolerance of $\epsilon =0.01$ is used in LS-OPT. This is more realistic for a direct FEM-based optimization strategies in comparison to solve an optimization problem for a pure analytical function like a surrogate model. However, the objective error of the verification FEM run for OD shows a clearly lower value as the default termination value and is sstill close to the optimum of the surrogate model.

#### Analysis of optimal design and selected designs

To obtain an sneak preview on the overall impact of changes in the objective function due to variations in the individual muscles pre-stretch parameters, i.e. sensitivity with respect to the optimal design, we analyse and compare the OD with three additional DPs. These three DPs distinguish themselves from the OD in a sense that these have been chosen from the set of 150 pre-defined DPs to evaluate objective function, joint motion and muscle forces acting on the joint. These specific DPs have been carefully handpicked such that they exhibit objective function values close to the OD and they are belonging to the dark blue optimal solution path (see Fig. 9). The objective values for all chosen design points are well below the tolerance limit of $1\,\%$ determined in respect to the experimental value ${\tilde{r}}$ and, therewith, they have the potential to be an optimal design, too. This can be seen by inspecting and comparing the objective values from the surrogate model (predicted), $F^{{}}_{\text{SM}}$, with the respective ones obtained by the finite element model, i.e. $F^{{}}_{\text{FM}}$. The respective values are presented in Table 6.

**Table 6:** Parameter set of initial muscle fibre pre-stretches of the optimal design (OD), $\textbf{s}^{\text{opt}}_{{}}$, and the three arbitrary handpicked design points (DP) with objective function values close to 0

	*m. anconeus*	*m. brachialis*	*m. biceps brachii*	*m. brachiradialis*	*m. triceps brachii*	$M_E$ [Nmm]	$F^{ {}}_{\text{SM}}$	$F^{{}}_{\text{FM}}$
OD	0.41137	0.35413	0.1574	0.19412	0.4252	126.0204	$1.076e-9$	$8.457e-3$
DP 8	0.0795	0.31463	0.12086	0.18651	0.40189	90.7354	$6.486e-3$	$2.844e-3$
DP 13	0.30814	0.30271	0.14965	0.20372	0.298668	$-$13.408	$5.092e-3$	$7.401e-4$
DP 147	0.08982	0.26634	0.15643	0.3265	0.31665	4.1523	$1.266e-3$	$7.126e-3$

From Table 6, one first observes that the different fibre pre-stretch parameter of DPs lead to different initial arm rotations manifested by the resulting joint moment at the elbow, $M^{{}}_{{E}}$, which is applied in LS-2 to bring the arm back to the initial position.

Negative-valued $M_E$’s originate in cases in which the arm is rotated due to a muscle’s pre-stretch from its initial position into flexion. Positive values describe an arm movement that rotates from $\Theta ^{{}}_{\mathrm{ini}}$ into extension. The OD has, in comparison to all DPs, the highest $M_E$. With 126.02 Nmm, while DP 147 has the lowest positive value with 4.15 Nmm. Only one of the considered DPs exhibits a negative value ($-$13.41 Nmm; DP 13).

Generally, one observes from the two tables that the more dominant the fibre pre-stretch of the flexors or extensors, the stronger the rotation in the respective direction and the more back rotation moment $M_E$ is required (OD exhibits the strongest back rotation moment of all considered design points).

Further, all determined pre-stretch values for *m. triceps brachii* are for all DPs much higher than those of the *m. biceps brachii* and *m. brachialis*. In addition, the *m. triceps brachii* value at the OD was the largest one for all selected DPs. Even in case of DP 13, which resulted in a negative back rotation moment, the *m. triceps brachii* fibre pre-stretch value was much lower. As we (have to) consider the entire musculoskeletal system, the negative moment is also a realistic case as it describes a case, in which muscle fibre stretch values are equal for the *m. biceps brachii* and *m. brachialis*. This underpins the key message of Fig. 6 and the optimal solution path that the fibre stretch of the *m. triceps brachii* is for achieving a physiological range of motion a crucial role and as well it has to be balanced in respect to the values of the flexors to achieve an optimal solution for the given initial pre-stretch values.

At OD, the fibre pre-stretch of *m. brachialis* differs with respect to its smallest *m. brachialis* fibre pre-stretch values by $24.79\,\%$ (DP 147), while the other two design points deviate only by 11.54 % (DP8) and 14.25 % (DP13), respectively. In contrast, all initial fibre pre-stretch parameters of the *m. biceps brachii* are lower than $\Lambda _{\textsf {opt}}$. Further, the fibre pre-stretch values of *m. anconeus* vary extremely. This is due to the muscle’s limited impact on the overall joint angle (cf. also Fig. 6). In other words, an error-prone initial stretch value for the *m. anconeus* has little impact on the overall biomechanical behaviour of this particular musculoskeletal system. In contrary, the pre-stretch values for the *m. brachiradialis*, are—except for DP 147—about the same (ranging from 0.18651 to 0.20372). For DP 147, the value is 0.3265. This indicates that the *m. brachiradialis* has the potential of significantly impacting the outcome of the biomechanical musculoskeletal system simulations.

### Analysis of arm motion driven by muscle activation

After investigating the optimization procedure and its solution process, we next focus on the heterogeneous stress/strain-distributions of the optimal design, OD. Furthermore, based on the pre-stretches determined for the herein considered initial configuration, an activation-guided calculation of elbow motion (forward model) is presented, i.e. the resulting stresses and strains of the individual muscles. Further, a sequence of 30 muscle activation levels is employed to study the influence of the pre-stretch on individual muscles with respect to its elbow joint angle.

#### Muscle stress–strain analysis of the optimal design

While Sect. 3.1 focuses on the optimization itself, i.e. analysing the optimal design point as a result of step LS-2 of our proposed algorithm (cf. Sec. 2.3.2), we focus now on analysing the pre-stretch heterogeneity of a particular skeletal muscle system configuration. After investigating the pre-stretch of the OD, we analyse the outcome of LS-4 and LS-6, i.e. the muscle’s mechanical state after either fully activating the extensor or flexor muscles.

**Fig. 10:**
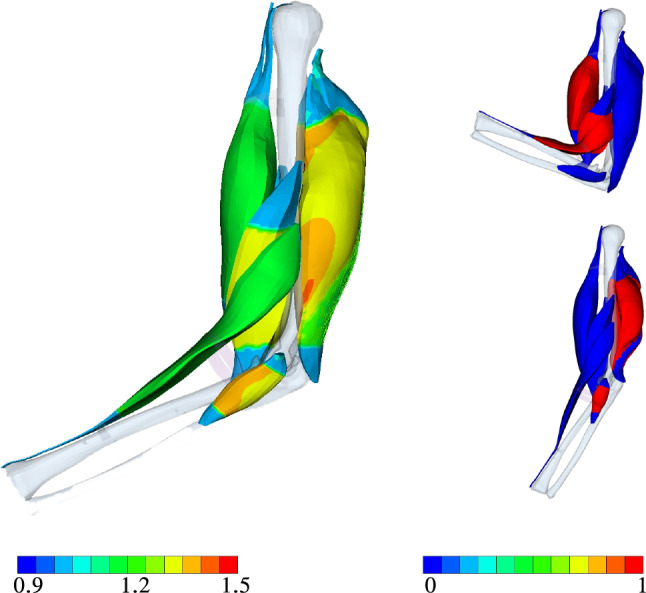
Left: Muscle fibre stretch $\bar{\Lambda }_{\text{S}}$ at OD after completing LS-2. Right: visualization of ${\varvec{\alpha }}^{{}}_{\text{Fl}}$ (top) and ${\varvec{\alpha }}_{\text{Ex}}$ (bottom). The gravity force is still working as illustrated in Fig. 3 even if the images are rotated by $270^{\circ }$

**Fig. 11:**
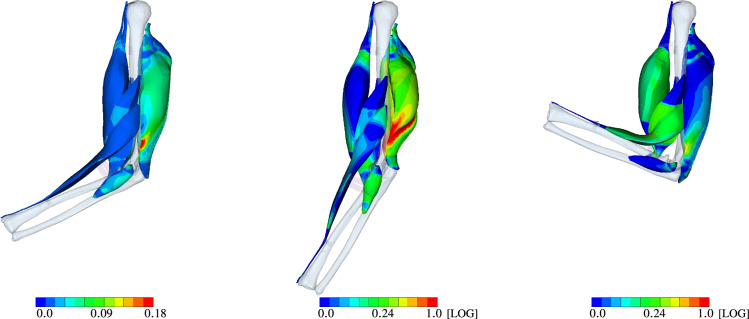
Lateral view on the equivalent *Green* strain of the musculoskeletal system after LS-2 (left), LS-4 (middle), and LS-6 (right)

Figure 10 depicts on the left the muscle fibre stretch $\bar{\Lambda }^{{}}_{\mathrm{S}}$ as obtained for OD after completing LS-2, while the figures on the right show the position and level of muscles activation during maximal extension and flexion. In the figures depicting the muscle activations, we drew material points that are fully contracting, i.e. $\alpha ^{{i}}_{{}}=1$, in red and the non-active parts (including the tendons), $\alpha ^{{i}}_{{}}=0$, in blue. Despite choosing a homogeneous and constant initial pre-stretch value for our algorithm proposed in Sec. 2.3.2, we obtain as a result non-homogeneous ones (cf. Fig. 10). This is due to computing the equilibrium steady state of the elbow joint after applying the initial muscle fibre pre-stretches and the following rotation of the musculoskeletal system back to its initial configuration.

The corresponding *Green* strain after back rotation (LS-2), after fully activating all extensors (LS-4), and after fully activating all flexors (LS-6) are shown in Fig. 11. Based on Fig. 11 (middle), i. e. LS-4, *m. triceps brachii* locally exhibits high strain concentrations. These regions correspond well with the location of the aponeurosis. During the flexion *m. biceps brachii* and *m. brachialis* are bulging due to muscle contraction with the underlying *m. brachiradialis* pushing up the *m. biceps brachii*. Without an additional and non-physiological elastic band, *m. brachiradialis* would be unnaturally straightened by the activation. Note, this added support structure on the joint system would not be needed, if all the surrounding muscles as well as the skin and collagenous structures would be considered in the model as well.

Further, Fig. 11 (left) shows that the equivalent *Green* strain of approximately $10\%$ (greenish color) is much higher for *m. triceps brachii* compared to the flexors after the pre-stretch optimization. There, local strains of up to 18% are predicted. As mentioned earlier, they are located at the transition between muscle tissue and the aponeurosis. A similar pattern can be observed for maximally activated extensors (Fig. 11 middle, after LS-4), and flexors (Fig. 11 right, after LS-6). In particular for extension subject to maximal activation, much higher strains are predicted than for the maximally flexion or the non-active, optimal pre-stretch case. At some locations within *m. triceps brachii*, they reach strain gradients of more than 100%. Hence, a logarithmic (LOG) scale is used to plot Fig. 11 (middle) and (right).

**Fig. 12:**
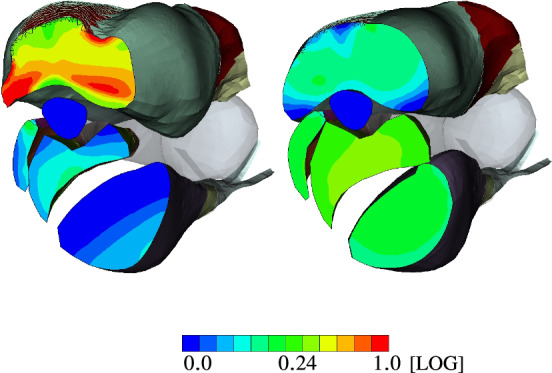
Green strain at LS-4 (left) and LS-6 (right)

**Fig. 13:**
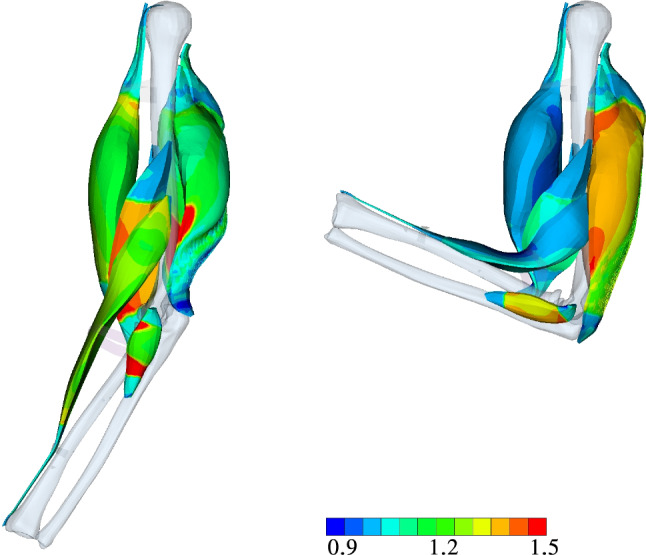
Muscle fibre stretch $\Lambda_{\text {S}}$ at LS-4 (left) and LS-6 (right)

**Fig. 14:**
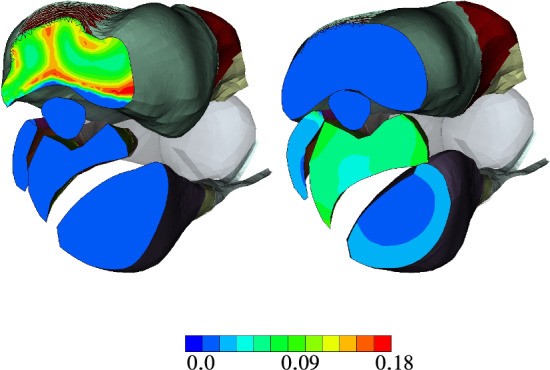
Active fibre stress $\mathit{S}^{\mathrm{ff}}_{\mathrm{act}}$ [MPa] at LS-4 (left) and LS-6 (right)

A cross-sectional view, as, for example given in Fig. 12, further highlights the heterogeneity in muscle deformations and the need for modelling skeletal muscles in a three-dimensional continuum–mechanical setting. Based on Fig. 12, the latter is essential for realistic analysis of muscle forces and thus the prediction of motion due to activation (forward problem). Figure 13 depicts the muscle fibre stretch and their corresponding active fibre stresses are shown in Fig. 14 (left) subject to maximally activated extensors, i. e. ${\varvec{\alpha }}^{{}}_{\mathrm{Ex}}$, and (right) maximally activated flexors, i. e. ${\varvec{\alpha }}^{{}}_{\mathrm{Fl}}$, respectively.

Figure 13 underpins the interply between the agonist and antagonist muscles within a musculoskeletal system. As the agonist lengthens the antagonist, the muscle fibre stretch in the agonist is reduced and vice versa. This directly impacts the stress generating abilities, which are length dependent. Further, the muscle fibres of the *m. triceps brachii* located close to the distal end of the humerus are stretched more than those towards the proximal side. Similar, the fibres located closer to the bone are more stretched than those further away. In particular the latter observation can be nicely seen in the cross-sectional view of the active stresses and muscle fibre stretch of the muscles as depicted in Figs. 13 and  14, respectively.

The active stresses, ${S}^{\mathrm{\text{ff}}}_{\mathrm {\text{act}}}$, of the flexors and extensors along the muscle fibre direction (ff) are below the maximally assumed stress, i.e. below $\sigma _{\textsf {max}}=0.3\,$ MPa (*Cauchy* stress) or in case of the 2nd Piola-Kirchhoff stress ${S}^{\mathrm {\text{ff}}}_{\mathrm {\text{act}}}=0.178$ MPa. This means that the force-generating potential of the muscle is not fully exploited for $\alpha =1$, which is only reached at the optimal fibre length. However, each arm position is only a snapshot of a deformation state at a material point within the muscles. The arm instantly rotates through contraction and the fibre length chances, too. Full muscle fibre contraction does not mean that the maximal potential active force of the muscle can be generated. In general, the active stress of OD within the *m. triceps brachii* is during maximal contractions of the extensor higher than, for example in the *m. biceps brachii* during maximal flexion. Within the *m. triceps brachii*, some of the active stress values almost reach in some region values the maximum.

**Fig. 15:**
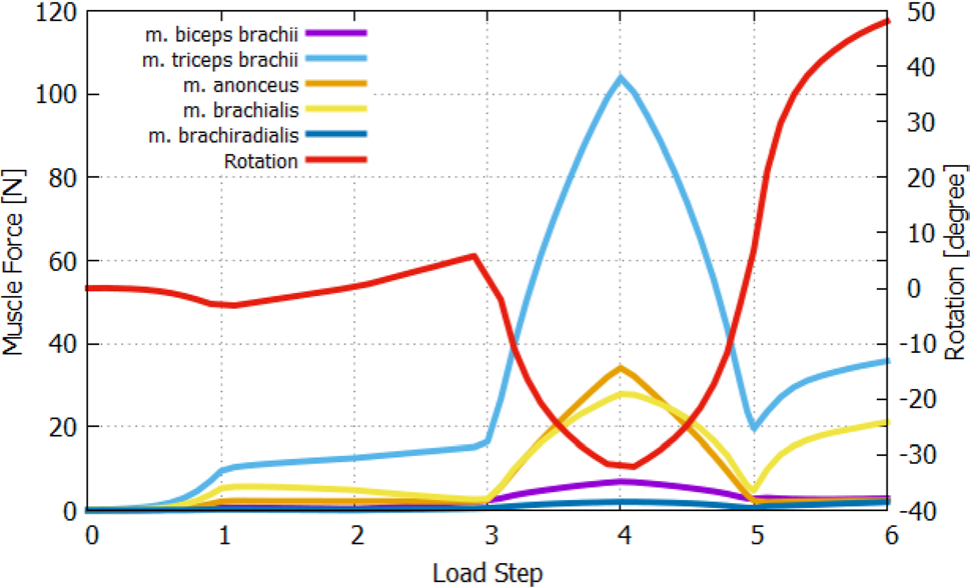
The muscle forces and arm rotation in respect to LS. Positive values represent a flexion movement and negative values an extension movement

Figure 15 aims to summarizes the entire process. It presents the exerted muscle forces (left y-axis) and arm rotation (red curve and right y-axis) in relation to the load steps. The different load steps are given the target of action level of each muscle, which have to be reached by linear increase in the muscle activation. Of course, each equilibrium state of the Newton iteration, which is needed for solving the nonlinear problem, is obviously an intermediate state. Accordingly, these intermediate states are also belonging to a specific muscle activation level. After LS-1, providing the system with general but constant pre-stretch value, induces a small arm extension, which is reversed to zero rotation at LS-2. In LS-3, the gravitational force causes a further small arm flexion. The amount of rotation depends on the passive joint stiffness due to the muscle’s pre-stretching. By executing LS-1, LS-2, and LS-3, the passive muscle forces are slightly changing. At LS-3, the passive muscle force of *m. triceps brachii* with 16.5 N is much higher than of the other muscle, which are less than 5 N. Due to the arm rotates to extension caused by the muslce’s fibre pre-stretch, the passive force of the extensors, in particular, of *m. triceps brachii* increases further in LS-2 and LS-3, while of the flexors decreases. Of the overall analysis, the *m. triceps brachii* exhibits the largest muscle forces at each load step. The muscle force of *m. anconeus* is high, but its influence to the flexion is neglectable small. The *m. triceps brachii* generates the most active muscle force in LS-4 about 103 N, because it is the only considered muscle that is responsible for extension compared to the activation of the flexors in LS-4 (*m. biceps brachii*: 2.8 N, *m. brachialis*: 21.1 N and *m. brachiradialis*: 2 N). A clear differences in passive force generation can observed of flexors and extensors if they behave as an antagonist. The *m. triceps brachii* generates very high resistance against flexion in LS-6 due to 35.8 N, while the passive force in *m. brachialis* against extension motion in LS-4 amounts 27.8 N and the sum of the flexors result to 36.4 N.

For our case, the resulting arm rotation is $-$32.2^∘^ in extension (LS-4) and 48.1^∘^ in flexion (LS-6). In total, this results in an absolute RoM of 80.2^∘^. Obviously, the $\sigma _{\textsf {max}}$ with a value of 0.3 MN, which is determined on a frog muscle, are not corresponding to human muscles and particularly not to each arm muscle. We are beware of the fact that $\sigma _{\textsf {max}}$ should be determined via isometric maximal contraction tests for each muscle of the human arm. This can be also approximately determined with an in-silico study using the OD. Nevertheless, the flexion ratio *r* used for the muscle fibre pre-stretch optimization is unaffected from the amount of RoM at the joint, but rather from the reached flexion and extension values in regard to each other. Regarding the individual DPs, all of them reaches a different RoM depending on the pre-stretch values. Here we have a load case that relates only to very small weights, namely the movement of the forearm against gravity. With the optimized muscle’s fibre pre-stretch in the hand, newly determined $\sigma _\textsf {max}$ values can be take from an experimental or an in-silico study. With the adjusted maximal isometric contraction forces of each muscle, any more complex forward musculoskeletal analysis can be done. However, the activation parameter for $\alpha =1$ needs to be newly calibrated to the corresponding $\sigma _\textsf {max}$ of each muscle.

#### Activation-driven forward simulations for analysis of muscle activity and motion

To better understand the relationships of muscle pre-stretch on muscle force development and resulting arm rotation, 30 activation scenarios have been simulated in a sequential way. These activation scenarios, AS, are combinations of individual muscle activations, $\upalpha ^{\mathrm {i}}_{{}} \in \{0,1\}$, of the respective five muscles. The activation or deactivation of the muscles up to to the next individual activation scenario are linearly increased or decreased, respectively. The relative changes of the arm rotation of succeeding scenarios are expressed by $\Delta \Theta$. Here, positive values of $\Delta \Theta$ represent a flexion and negative ones an extension in regard to the last arm position. The activation scenarios and $\Delta \Theta$ are listed in Table 7. Note, the first three activation scenarios are the base load cases for initializing the musculoskeletal system boundary value problem of the OD. These include setting up the muscle fibre pre-stretch (LS-1), back rotation to its initial state (LS-2) and considering gravitational force (LS-3).

Then, we first activate the *m. biceps brachii* (AS 4) followed by additionally activating *m. brachialis* and *m. brachiradialis*, AS 5 and AS 6, respectively, whereas AS 6 corresponds to LS-6. The lastly activated *m. brachiradialis* (AS 6) does not contribute much to the flexion with $1.2^{\circ }$ in regard to the joint position at AS 5, while *m. biceps brachii* and *m. brachialis* are successively generating $19^{\circ }$ and $19.9^{\circ }$ of flexion. Figure  17 shows the muscle forces of *m. biceps brachii*. It amounts to 13 N at AS 4 and decreases to 3 N due to the activation of *m. brachialis* at AS 5, while the muscle force of *m. brachialis* shown in Fig. 18 increases from 2.2 N to 21.1 N. With increasing of flexion by activating the *m. brachialis*, the muscle force of *m. biceps brachii* drops due to further reduction of the muscle fibre stretch. Consequently, the muscle force of *m. biceps brachii* decreases. The reason for the low contribution of *m. brachiradialis* to motion is the low muscle fibre pre-stretch since the initial arm position is in a flexed state. There is also the additional fact that the arm is already flexed due to the two active flexors when *m. brachiradialis* starts getting activated. This leads to further shortening of the muscle fibres of *m. brachiradialis*.

However, in AS 30, only *m. triceps brachii* is activated, as the elbow joint is already in extension. In this position, the *m. brachiradialis* is stretched (AS 31) in such a way that it can produce significant force to change the elbow angle by $28.76^{\circ }$ due to elongated muscle fibres of *m. brachiradialis*. As seen in Fig. 15, *m. anconeus* produces 34 N of active force in LS-4. This, however, does not contribute to a joint moment, compared to AS 30 where only *m. triceps brachii* is activated. The difference in rotation amounts to $2^{\circ }$. This is likely due to its smaller lever arm with respect to the joint.

**Table 7:** Muscle activation scenarios (AS) and resulting change in elbow angle $\Delta \Theta$ with respect to the previous AS. Negative $\Delta \Theta$ values resembles extension and positive values flexion

Muscle	m.bi	m.ba	m.br	m.an	m.tr	$\Delta \Theta$
AS 1 (LS-1)	0	0	0	0	0	$-3.07$
AS 2 (LS-2)	0	0	0	0	0	3.39
AS 3 (LS-3)	0	0	0	0	0	7, 64
AS 4	1	0	0	0	0	19
AS 5	1	1	0	0	0	19.9
AS 6 (LS-6)	1	1	1	0	0	1.2
AS 7	1	1	1	1	0	$-4.87$
AS 8	1	1	1	1	1	$-11.3$
AS 9	1	1	1	0	1	$-7.95$
AS 10	1	1	0	0	1	$-1.47$
AS 11	1	1	0	1	1	$-0.91$
AS 12	1	1	0	1	0	10.4
AS 13	1	0	0	1	0	$-15.7$
AS 14	1	0	1	1	0	$-3.62$
AS 15	1	0	1	1	1	$-18.3$
AS 16	1	0	1	0	1	$-1.31$
AS 17	1	0	1	0	0	16.7
AS 18	0	0	1	0	0	$-3.24$
AS 19	0	1	1	0	0	23.3
AS 20	0	1	1	1	0	$-2.76$
AS 21	0	1	1	1	1	$-11.1$
AS 22	0	1	1	0	1	$-1.99$
AS 23	0	1	0	0	1	$-0.51$
AS 24	0	1	0	0	0	13.5
AS 25	0	1	0	1	0	$-5.38$
AS 26	0	1	0	1	1	$-15.1$
AS 27 (LS-4)	0	0	0	1	1	$-57.7$
AS 28	1	0	0	1	1	20.6
AS 29	1	0	0	0	1	$-0.94$
AS 30	0	0	0	0	1	$-21.2$
AS 31	0	0	1	0	1	7.56
AS 32	0	0	1	1	1	2.76
AS 33	0	0	1	1	0	35.8

**Fig. 16:**
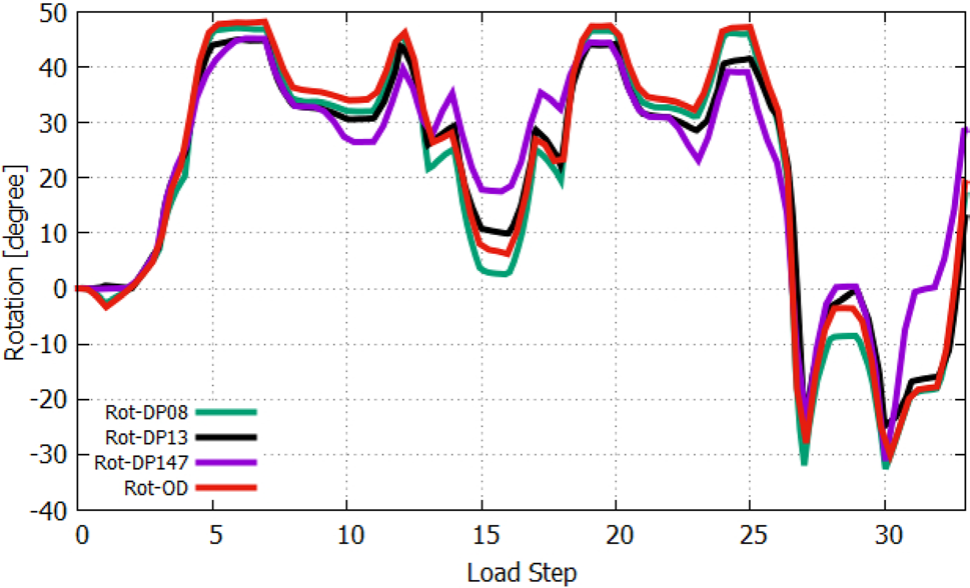
The arm rotation in respect to LS of selected DPs and OD

Next, we investigate the impact of the pre-stretch on the musculoskeletal system’s range of motion. In this regard, we use the previously handpicked DP again that exhibited objective function values close to OD, i.e. zero. The results for DP 8, DP 13, DP 147, and OD are shown in Fig. 16.

From Fig. 16 one clearly observes that the pre-stretch has a significant impact on the overall elbow range of motion. Even small deviations to pre-stretches with nearly the same optimization function value can differ up to $20^\circ$ for specific activation scenarios. Due to the high muscle fibre pre-stretches of *m. biceps brachii* and *m. triceps brachii*, OD generates the maximal extension and flexion values and, hence, achieves the largest RoM. This is followed by DP 8 that exhibits a slightly lower initial stretch values in the *m. brachialis*.

**Fig. 17:**
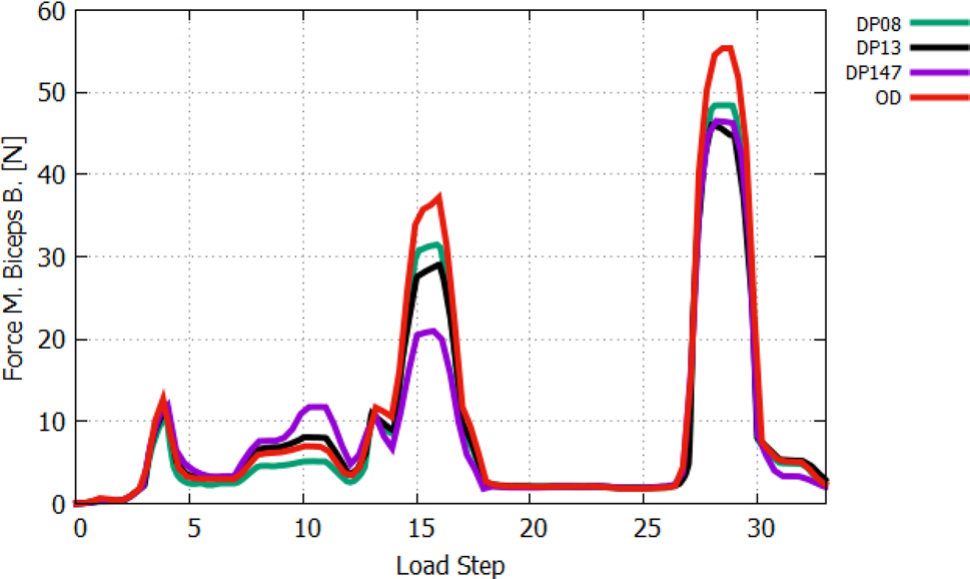
The *m. biceps brachii* forces in respect to LS

**Fig. 18:**
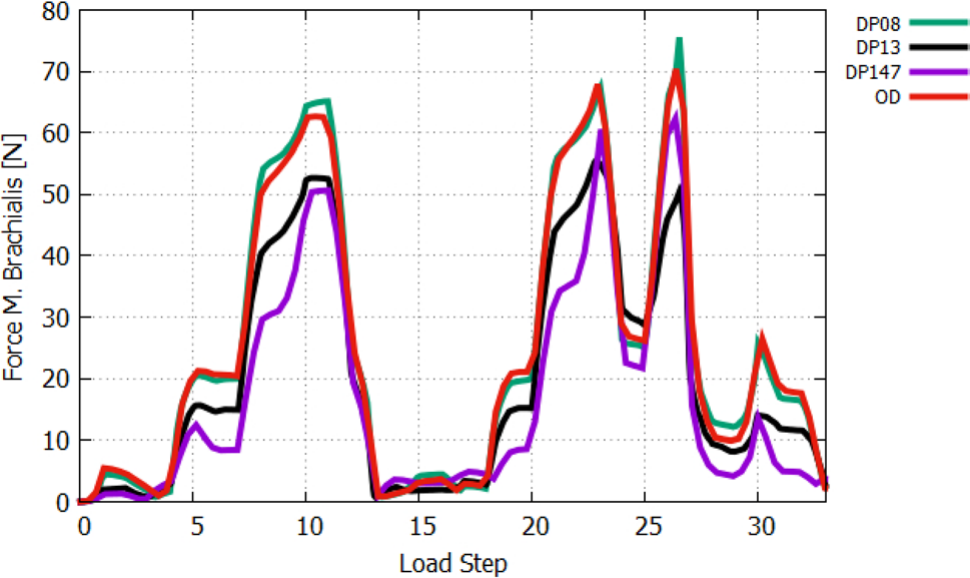
The *m. brachialis* forces in respect to LS

**Fig. 19:**
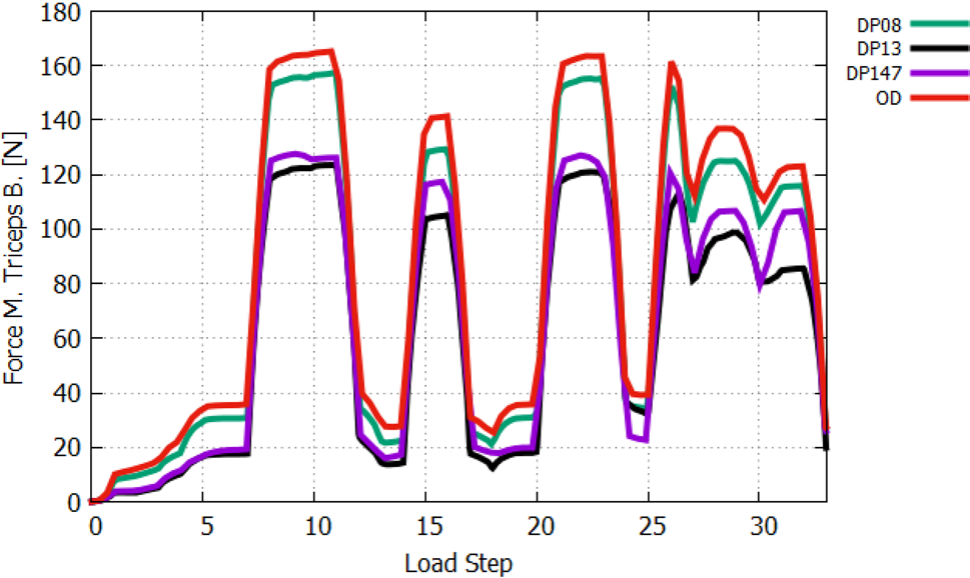
The *m. triceps brachii* forces in respect to LS

The respective generated muscle forces for the *m. biceps brachii*, *m. brachialis* and *m. triceps brachii* are given for the different design points in Figs. 17, 18, and 19, respectively.

The muscle forces of *m. brachialis* can be as high as 62.4 N for AS 11, 67.4 N as for AS 23 and 70 N as for AS 26, while *m. biceps brachii* has the highest muscle forces for AS 16 of 38 N and at AS 29 of 55.5 N. In all these cases, *m. triceps brachii* is also active and generates the highest muscle forces too with AS 11=165 N, AS 16=141.6 N, AS 23=163.8 N and AS 26=161.5 N. Such high muscle forces are developed because they mutually impede the fibre shortening due flexion or extension motion. Hence, more muscle forces can be produced with less motion. For these activation scenarios, the arm flexion angle $\Theta_{{\text{Fl}}}$ amounts to $35^{\circ }$, $32.2^{\circ }$ and $32.7^{\circ }$ for AS 11, AS 23 and AS 26, respectively. The elbow angle values are at the same level. These cases, where agonists and antagonists are simultaneously active, are less physiological in a healthy person, but in spacticity could be occur similar co-contractions. The imbalance in muscle activity impedes muscle relaxation after contraction and stiffens the joint. Generally, when agonist muscles are active to generate motion, the antagonist muscles work like a passive resistance, i.e. the passive forces increase slowly but exponentially with the elongation of muscle fibres. Therewith, the rate of resistance against motion is higher at higher fibre stretches. The maximal flexion of $48.1^{\circ }$ with active flexors needs less muscle forces of 26.4 N, while the active extensors generates maximal extension upto $-32.2^{\circ }$ with 137.9 N. The contribution of *m. brachiradialis* and *m. anconeus* is 2.65 N and 35 N, respectively, without having much on the overall motion. If we compare the muscle force of DPs with OD, the relative difference between the different force levels is up to 40 $\%$ for *m. biceps brachii*, 31 $\%$ for *m. brachialis* and 25 $\%$ for *m. triceps brachii*, even though the objective function value of the DPs are in a similar low range, i.e., less than 1$\,\%$.

## Discussion

The need for appropriate pre-stretches is not only a challenge for continuum–mechanical skeletal muscle modelling frameworks, but also for multi-body ones. Despite the significantly larger body of literature reporting on multi-body musculoskeletal system models, there also seems to be a lack of appropriately determining pre-stretch. Mörl et al. (2020), for example, assume that the rest length of the elastic element parallel to the contractile element is a constant factor of the respective muscles’ optimal length of the contractile element. Choosing a factor of 0.95 (Mörl et al. 2020) relates to an average pre-stretch of 5 %. The choice of pre-stretch, albeit a significant contributor to the overall exerted forces, is based on expectations and experience and is typically chosen to be constant across all muscle-tendon units.

Due to the three-dimensional structural behaviour of the muscles individually and in a complex system, an idealization to a 1D model for a multi-body analysis is only possible under strong simplification. This effect becomes even more obvious, where large rotation (geometric nonlinearity) is triggered by muscle activation and leads to increased inhomogeneous stress and deformation states. However, challenging part in 3D muscle modelling is not only the geometry of the muscles and tendons but rather of ligaments covering the tendons at the joint and fat and skin covering the muscles to kept them close to the joint and bone structure. In particular, the ligaments are important to stabilize the joint in order to keep all in position for efficient work of the joint. Therewith, the hinge joint with axial rotation of the lower arm could be modelled without defining particular boundary conditions with fixing the degrees of freedom. Using such an unconstrainted elbow joint, a pronation and supination of the elbow could be also simulated.

The correct choice of weighting parameters for achieving the best possible maximization of the objective functions depends on the biomechanical parameters, which are person-specific. These parameters are, for example, the RoM of the joint, muscle activation potential, load capacity, the neutral position of the joint, which can change due to muscle growth or degradation, and many others. Through the presented optimization process, static and dynamic joint stiffnesses can be determined person-specifically, especially for dysfunctional musculoskeletal joints. This would make it possible, for example, to develop and design orthoses, prostheses or active therapeutic exoskeletons specifically to the required therapeutic support for the individual biomechanical joint behaviour and to stimulate muscle training in a positive way. But also physics-based planning and control of muscle and tendon transfers and tissue balancing in joint related interventions such as joint implantations or major amputations may profit.

Modelling of skin and fat would be more relevant for analysis of externally interacting structures. Therewith, the dynamic external loads can cause soft tissue deformations and damage and influence the muscle performance too. The structure of such protectors or sockets could be analysed in order to adapt their construction better and more target-oriented to the person-specific musculoskeletal performance. More relevant application using musculoskeletal analyse is for personalized orthoses. They can only work effectively if the orthosis function is exactly adapted to the passive and actively generated joint stiffnesses. The interaction forces between the limb and orthosis are generated by the muscle forces. The right person-specific forces are important to design the functionality of orthosis structure individually, otherwise the limb will work against it without any therapeutic aid.

Based on the optimization strategy presented for determining of the complex 3D musculoskeletal systems, an optimization strategy must also be developed for determining an energy-efficient muscle activation path for all muscles that are involved. The goal here must be to activate each muscle when it is effectively providing muscle force in a particular arm position, which then leads to arm movement. In other arm positions, the muscle would otherwise work inefficiently or, in the worst case, counterproductively and against the movement, thus providing resistance. In the case of dynamic rotation (very fast movement), the rotating forearm must be decelerated to stop the movement exactly in the target position. This task is performed by the antagonists by generating the appropriate deceleration force through a dosed muscle activation. The determination of the energy-efficient muscle activation strategy for static and dynamic cases has to be done via a computationally efficient sensitivity analysis of different activation patterns $\alpha \ge 0$ of the muscles. Based on the data, an optimization strategy can be developed and investigated via surrogate models under the target of suitable rotation.

Since there is no one optimal design, but a variety of optimal solutions are possible, additional dynamic measurements of active muscles e.g. with ultrasound or dynamic MRI could help to determine individual muscle length changes or muscle fibre changes in the movement. This measurement data would be included as input data in the optimization of the muscle fibre and restrict the solution space.

## Conclusion

In conclusion, we were able to show that pre-stretching of muscle fibres in a 3D multi-muscle model has a major influence on the movement of the elbow joint in flexion and extension. The levels of these pre-stretches cannot be readily measured in vivo. FE simulations were carried out on a detailed arm model with three flexors and two extensors. This enables the simulation of joint movement in both directions, i.e. flexion and extension. By analysing the sensitivity of muscle pre-stretches on joint movement, we demonstrated that simulation of joint kinematics provides reliable results only if the agonist and antagonist muscles are modelled together with appropriate pre-stretch levels. Not only does the passive stiffness depend on the muscular pre-stretch, but also the active muscle force development for generation of motion relies heavily on it. By introducing the optimization procedure for determining muscle fibre pre-stretches, we were able to show that the objective function, defined as the ratio of flexion and extension with respect to the RoM, is suitable for calibrating muscle pre-stretches for flexion and extension behaviour to the measured movement. In addition, we were able to demonstrate how muscle pre-stretch affects the arm movement by means of a muscle activation study with different $\alpha$ values. Results of this study were presented for the optimal design and three other design points from the numerical optimization, which also fulfilled the objective criterion of the flexion/extension ratio. In this study, we could show that variations in the pre-stretch levels of individual muscles within multi-muscle models results in different elbow movements.

### Limitations

Anatomical joints do not have fixed joint axes for rotation and translation. Their physiology is guided and, to a certain extent, limited by pre-stressed connective tissues and muscle-tendon structures. Bone morphology and cartilage mechanics also influence the motion trajectories of bone segments. Additionally, connective tissue structures stabilize the joint. Strict limitations of standard joint modelling approaches, known from mechanical engineering and used in 1D MBS with muscles, thus, do not represent the complex physiological motion of human joints. Therefore, the next step is to re-model the additional anatomical structures of the elbow joint without constraints and with connective tissues in order to simulate their physiological function even more realistically. In terms of soft tissue properties, the material and structural stiffness transitions gradually from muscle-specific to tendon-specific properties, which has been approximated using a discrete transition zone. A non-local material formulation would give a more accurate representation of the deformation behaviour.

For clinical applications, it is necessary to have musculoskeletal models with joints representing accurate physiology without any constraints in order to simulate predictable changes of joint kinematics through surgical interventions. For example, patients suffering from brachial plexus injury eventually lose their elbow function and need to undergo a plastic reconstruction of the elbow function. Such reconstruction techniques involve a tendon or free muscle transfer, which has to be sutured with an appropriate muscle tension to ensure that the resulting joint motion is as optimal as possible. For the success of such an operation, the stretch of the transferred muscle or tendon to generate enough muscle forces and ensure correct joint movement is essential (Chuang et al. 1996; Gohritz et al. 2008).

The current simulations have been carried out for quasi-static load-cases. Inertial terms have been neglected in order to study and understand the slow motion of musculoskeletal limb. The initialization of the muscle pre-stretch, itself, is independent of the velocity of the activation or dynamic motion of the limb, as it is prevalent primarily under static equilibrium conditions. However, typical human movements involve more dynamic aspects, such as limb acceleration and deceleration, are requiring the integration of dynamic terms into the model.

General estimations of parameter values of soft tissue models is not possible as they are strictly subject-specific. Such parameters vary strongly based on physiological and pathological conditions, including factors such as genetics, age, training status of the muscle and many more. There is also the fact that their values are very difficult to identify from in-vivo experiments, which is only possible with non-invasive measurements or cadaveric tissues test with high inaccuracy, simplification and falsification. In consequence, an accurate and rigorous verification and validation of the mechanical properties of soft tissues is not possible without reliable experimental data sets.

## A Glossary

See Table 8.

**Table 8:** Abbreviations glossary

ANISO	-	Anisotropy
ANOVA	-	Analysis of variance
AS	-	Activation scenario
CAD	-	Computer-aided design
CT	-	Computed tomography
DP	-	Design point
DTI	-	Diffusion tensor imaging
Ex	-	Extension
FEM	-	Finite element method
Fl	-	Flexion
FM	-	Finite (element) model
GSA	-	Global sensitivity analysis
ISO	-	Isotropy
LS	-	Load step
MBS	-	Multi-body system
MRI	-	Magnetic resonance imaging
PC	-	Personal computer
RMS	-	Root-mean-square
RoM	-	Range of motion
SQP	-	Sequential quadratic programming
SM	-	Surrogate model
1D or 3D	-	One-dimensional or three-dimensional
Subscript act	-	Active
Subscript asc	-	Ascending
Superscript ba	-	*m. brachialis*
Superscript bi	-	*m. biceps brachii*
Subscript dsc	-	Descending
Subscript ini	-	Initial
Superscript *M*	-	Muscle
Superscript *MTZ*	-	Muscle–Tendon-zone
Subscript opt	-	Optimum
Superscript *T*	-	Tendon
Subscript pas	-	Passive
Superscript sim	-	Simulation
Superscript tr	-	*m. triceps brachii*

## Footnotes

1 LS-OPT is a standalone Design Optimization and Proba­bilis­tic Analy­sis pack­age with an in­ter­face to LS-DY­NA.
